# One Health Action against Human Fascioliasis in the Bolivian Altiplano: Food, Water, Housing, Behavioural Traditions, Social Aspects, and Livestock Management Linked to Disease Transmission and Infection Sources

**DOI:** 10.3390/ijerph19031120

**Published:** 2022-01-20

**Authors:** René Angles, Paola Buchon, M. Adela Valero, M. Dolores Bargues, Santiago Mas-Coma

**Affiliations:** 1 Cátedra de Parasitología, Facultad de Medicina, Universidad Mayor de San Andrés (UMSA), Av. Saavedra, Miraflores, La Paz 10077, Bolivia; anglesrene@hotmail.es; 2 Unidad de Limnología, Instituto de Ecología, Universidad Mayor de San Andrés (UMSA), Campus Calle 27, Cota Cota, La Paz 10077, Bolivia; pbuchon31@gmail.com; 3 Departamento de Parasitologia, Facultad de Farmacia, Universidad de Valencia, Av. Vicent Andres Estelles s/n, 46100 Burjassot, Valencia, Spain; Madela.Valero@uv.es (M.A.V.); S.Mas.Coma@uv.es (S.M.-C.)

**Keywords:** human and animal fascioliasis, Northern Bolivian Altiplano hyperendemic, One Health action, infancy and gender problems, transmission foci, food and water infection sources, household and knowledge, behavioural, traditional, social aspects, livestock management, prevention and control

## Abstract

The Northern Bolivian Altiplano is the fascioliasis endemic area with the reported highest human prevalence and intensities. A multidisciplinary One Health initiative was implemented to decrease infection/reinfection rates detected by periodic monitoring between the ongoing yearly preventive chemotherapy campaigns. Within a One Health axis, the information obtained throughout 35 years of field work on transmission foci and affected rural schools and communities/villages is analysed. Aspects linked to human infection risk are quantified, including: (1) geographical extent of the endemic area, its dynamics, municipalities affected, and its high strategic importance; (2) human population at risk, community development and mortality rates, with emphasis on problems in infancy and gender; (3) characteristics of the freshwater collections inhabited by lymnaeid snail vectors and constituting transmission foci; (4) food infection sources, including population surveys with questionnaire and reference to the most risky edible plant species; (5) water infection sources; (6) household characteristics; (7) knowledge of the inhabitants on *Fasciola hepatica* and the disease; (8) behavioural, traditional, social, and religious aspects; (9) livestock management. This is the widest and deepest study of this kind ever performed. Results highlight prevention and control difficulties where inhabitants follow century-old behaviours, traditions, and beliefs. Intervention priorities are proposed and discussed.

## 1. Introduction

Fasciolid trematodes are helminth parasites characterized by their low specificity at the level of the definitive host. The infection of livestock species is of veterinary importance because of the big losses in husbandry they cause [1]. Their capacity to infect and develop in humans underlie a disease which may induce severe pathogenicity, sequelae, community underdevelopment, and even death [2,3] in rural areas of mainly low income countries, but also developed countries [4,5], in which long term sequelae have been observed in treated patients [6].

Two liver fluke species are the causal agents of this disease, differing in geographical distribution due to their specificity towards their freshwater snail vector species belonging to the family Lymnaeidae [7]. *Fasciola hepatica* is mainly transmitted by small amphibious species of the *Galba*/*Fossaria* group, whose wide distribution allowed this fasciolid to colonize cold temperate regions of all continents excepting the two poles. *Fasciola gigantica* is more pathogenic [8], although restricted to warm regions of only Africa and Asia, where it is transmitted by usually bigger, more aquatic lymnaeid species of the *Radix* group [9]. Thus, the absence of *Radix* throughout the Americas has recently been argued to constitute an insurmountable filter for the introduction of *F. gigantica* into the New World [10].

The transmission patterns and the epidemiological characteristics of this disease are in great part defined by the ecological requirements of these freshwater snail vectors. The marked susceptibility of lymnaeids to the habitat features explain the pronounced influences of climate change and anthropogenic modifications of the environment on this disease, at the level of both animals [11] and humans [12]. These changes, together with the human infection sources, underlie the recent emergence of this disease in many areas. Human fascioliasis infection sources have recently been re-assessed at a worldwide level and have demonstrated that (i) there are sources which had not be considered before, (ii) they differ according to local areas and countries, and (iii) they include foods, water, and combinations of both [13]. The following should be listed as sources for the ingestion of metacercariae: freshwater and semi-aquatic plants, but also terrestrial plants needing frequent irrigation, traditional local dishes made from sylvatic plants, and vegetables sold in uncontrolled urban markets; contaminated natural water drinking, and beverages, juices, soups, and dishes made with natural water; as well as the washing of kitchen utensils, vegetables, fruits, and tubercles with contaminated natural water [13].

The aforementioned aspects of pathogenicity and the increasing number of human case reports and new human endemic areas in which children appear to be the most infected, and the immunosuppression effect during the long chronic phase of this disease [14] related to many coinfections with other pathogenic protozoan and helminthic diseases, led the World Health Organization (WHO) to include human fascioliasis within the priority list of Neglected Tropical Diseases (NTDs) for the 2010–2020 period [15]. Fascioliasis is the only disease showing worldwide distribution among the NTD group of food-borne trematodiases, which has been again included in the NTD Roadmap of WHO for the period up to 2030 [16].

Fascioliasis is an important public health problem in many countries of the Americas, from Mexico in the North [17] up to Chile [18,19] and Argentina [20,21] in the South. Worth mentioning are the high human prevalence and intensity reported in high altitude areas of Andean countries such as Venezuela [22], Peru in both valleys [23] and the altiplano [24], and Bolivia.

It is in Bolivia where the highest prevalence by both coprology [25,26,27] and serology [28,29,30], and also the highest intensities estimated by egg output quantification [9,26] have been reported. This epidemiological situation, with local prevalence up to 72% by coprology and 100% by serology, and local intensities up to more than 8000 eggs per gram of faeces (epg), appears restricted to the Northern Bolivian Altiplano. Children and females are the age and sex groups most affected in that human hyperendemic area, posing a community development problem [31] and a social problem concerning gender in the Aymara-inhabiting populations.

Moreover, similarly as in the other aforementioned Andean countries, human fascioliasis endemic areas concern high altitude areas, among which the Northern Bolivian Altiplano is the one located at the highest altitude, namely between 3820 and 4100 m a.s.l. [32]. The extreme environmental conditions at such a very high altitude have three crucial repercussions on the transmission and epidemiology of fascioliasis in the Bolivian Altiplano:

- Human inhabitants are also obliged to adapt to such extreme altitudinal rural environment; thus, they mainly depend on livestock, because plant cultures require a lot of effort and produce low, insufficient yields, useful only for subsistence; sheep, cattle, pigs, and donkeys are the main species owned by families, and the four species most infected by *F. hepatica* in that area [27];
- Climate characteristics including (i) the lack of marked differences of temperature throughout the whole year and (ii) the high altitudinal evapotranspiration [32] leading lymnaeid populations to mainly inhabit permanent freshwater collections, together with the long infection capacity kept by the metacercariae under low temperatures [33], assure a permanent transmission with human and animal infection risk throughout the whole year, i.e., lack of seasonality;
- The transmission is markedly enhanced when compared with lowlands [34], because (i) the infected snails follow a longer patent period, with a cercarial shedding process being twice as long, (ii) the production of cercariae by each snail is pronouncedly higher, (iii) the infected lymnaeids survive longer [35], and (iv) the uterus of the adult stage is shorter, favoring a continuous shedding [36,37].

This scenario gives rise to a great circulation of *F. hepatica* throughout the endemic area and explains the permanent infection risk for both humans and animals, as well as the high re-infection risk, which in turn increases egg shedding by humans and animals [38]. Indeed, there is no premunition during the chronic phase of this disease [39]. T cells have been seen to decrease their cytokine responses and enter into reduced proliferative activity in this phase, which underlies their hypo-responsiveness to antigen stimulation [40]. This leads to two additional problems: (i) fluke adult accumulate in re-infected subjects and the increasing burden becomes more pathogenic, and (ii) this immuno-suppression during the chronic phase facilitates coinfections with other protozoan and helminthic diseases [14], such coinfections becoming the rule in children [41,42], thus defining a scenario of high parasitic infection burdens in the rural human communities of the Altiplano.

Such a concerning health scenario led WHO to prioritize the Northern Bolivian Altiplano human hyperendemic area within the worldwide initiative against this disease. A pilot intervention in 2007 [43,44] allowed for the verification of the absence of secondary effects in the treatment of children with a low mono-dose of 10 mg/kg of triclabendazole for human use (Egaten^®^ donated by Novartis Pharma, Basel Switzerland) [45]. Subsequent campaigns of preventive chemotherapy by means of yearly mass treatments covering the whole endemic area were implemented thereafter with the objective to decrease morbidity by decreasing individual burdens. Monitoring results exposed in a specific liver fluke surveillance WHO/PAHO meeting in La Paz in 2014 showed the problem of infections and reinfections between the yearly campaigns, as a consequence of the high infection risk assured by the high infection rates at animal reservoir level and the distribution of numerous populations of the unique lymnaeid vector species *Galba truncatula* overall [46].

A complete One Health control initiative was decided to be implemented to complement the yearly preventive chemotherapy campaigns. The selected objectives were designed to reduce infection and re-infection risks, by acting on the key aspects of the disease transmission. Five main multidisciplinary complex axes were defined: (i) animal reservoirs; (ii) lymnaeid snail vectors; (iii) environment and climate; (iv) human infection; and (v) social aspects, behaviour, traditions, and other characteristics of the lifestyle of the humans inhabiting this very high altitude hyperendemic area.

This ongoing intervention has already furnished many applied results. The transmission capacity and reservoir role have been experimentally studied and surveyed in the field in the cases of all Altiplanic animal species which have been mentioned to be involved in the disease circulation. Sheep and cattle have demonstrated that they are able to participate as the main reservoirs, even at such a high altitude [47]. The domestic pig has proven to be the third most important reservoir, despite many previous studies arguing against the potential role of this animal in fascioliasis [48]. The donkey demonstrated that it was the fourth reservoir species involved, with a problematic role in the geographical diffusion of both liver fluke and snail vector [49]. The llama, previously suggested to participate in the disease transmission in the Altiplano, showed an insufficient transmission capacity and a specific defaecating behaviour which allowed us to neglect South American camelids as reservoirs and consequently indicated that there was no need to include them in control measures [50].

All Altiplanic lymnaeid vector populations were proven to belong to the species *G. truncatula*. DNA multi-marker sequencing showed that there is a monomorphic, genetically unique strain, which pronouncedly simplifies control measures because of their homogeneous ecology and ethology [46]. A wide environmental and climatic assessment of the distribution of lymnaeid snails demonstrated that most of the Altiplanic vector populations are linked to permanent freshwater collections [51]. However, recent field surveys of transmission foci showed that the endemic area is spreading thanks to an adaptation to higher altitudes. The new endemic zones detected imply a widening of the endemic area targeted for preventive chemotherapy and indicate the importance of considering the changing dynamics of the transmission risk area [46].

The present study concerns the aforementioned fifth axis. The aim is to analyse all characteristics of the lifestyle of the inhabitants of the Northern Bolivian Altiplano human hyperendemic area which may be linked to human infection or influence the transmission and epidemiology of fascioliasis. This multidisciplinary analysis of social aspects, behaviour, and traditions includes both (i) specific studies focused on key features such as food, edible vegetables, sources of water drinking, and livestock management, as well as (ii) an appropriate analysis of the information obtained throughout a long period of more than 35 years of field work on disease transmission foci, rural schools, and communities/villages in whose surveys infected subjects were detected. Two singularities need to be emphasized: the inhabitants belong to the Aymara ethnic, and their life is markedly conditioned by the extreme characteristics of the very high altitude of the endemic area. Aymaras transmit their knowledge orally; therefore, literature sources regarding the aforementioned aspects of interest are very few or even sometimes inexistent, although some scattered useful information has been found in several recent articles, mainly appearing in non-peer-reviewed sources, non-scientific local publications, and grey literature. All in all, a second purpose of this study is to offer an example which may be extrapolated to other human fascioliasis endemic areas about which factors should be considered, by which way to appropriately approach them, and how to perform an analytical description useful for the design of disease control measures.

## 2. Materials and Methods

### 2.1. Study Area

Field studies on fascioliasis in the Northern Bolivian Altiplano were launched in July 1983 and have been yearly followed until today. Initial steps were devoted to geographical prospections to assess the extent of the endemic area and delimitation of its external borders by three strategies:

- Assessing the distribution of lymnaeid snails allowing for liver fluke transmission: Lymnaeid snails were differentiated from other freshwater snails by their pair of triangular tentacles with darkly pigmented eyes at their bases and their typical small, smooth, and dextral conical shell. In the Northern Altiplano, there are no other snails in the freshwater collections which may be confused with lymnaeids [51]. To distinguish between lymnaeid species with liver fluke transmission capacity, i.e., lymnaeid intermediate hosts or vectors, and non-transmitting lymnaeid species potentially present because known to also inhabit Andean high altitude areas [22], lymnaeids from each population collected were molecularly characterized by the sequencing of two ribosomal DNA markers (ITS-2 and ITS-1) and two mitochondrial DNA markers (16S and *cox*1). These genetic analyses demonstrated that a single, genetically monomorphic lymnaeid vector species is present in the Northern Bolivian Altiplano and is the responsible of fascioliasis transmission in this endemic area, namely *Galba truncatula* [46].
- Analysing the geographical extent of fasciolid infection in cattle: Cattle was the livestock species selected for this assessment because (i) this ruminant reservoir is present and distributed throughout the Northern Bolivian Altiplano and has proved to significantly participate in the transmission of the disease [47]; (ii) it is the most important species among the livestock owned by each Altiplanic Aymara family [27]; and (iii) cattle is the most appropriate livestock species for such a geographical extent assessment because egg output in this animal only lasts for a short time, a high production of eggs has a duration of only a few weeks, resistance in this ruminant is acquired already from the first infection, and consequently fascioliasis in cattle is self-limiting with the majority of flukes being eliminated in the initial 9–12 months, a host–parasite interaction pronouncedly different from the long-lasting infection and egg shedding in sheep [9].
- Verifying that lymnaeid snail presence and cattle infection was geographically followed by human infection: To assess the geographical overlap of human infection with the distribution of the lymnaeid vector and cattle infection, liver fluke infection in 5–15-year-old schoolchildren was coprologically diagnosed [26], because (i) primary rural schools are present and distributed throughout the Northern Bolivian Altiplano, (ii) each one of these schools receives children from its surrounding zone, and (iii) faecal output of eggs indicates active infections, whereas serological tests do not necessarily indicate such [52].

**Figure 1:**
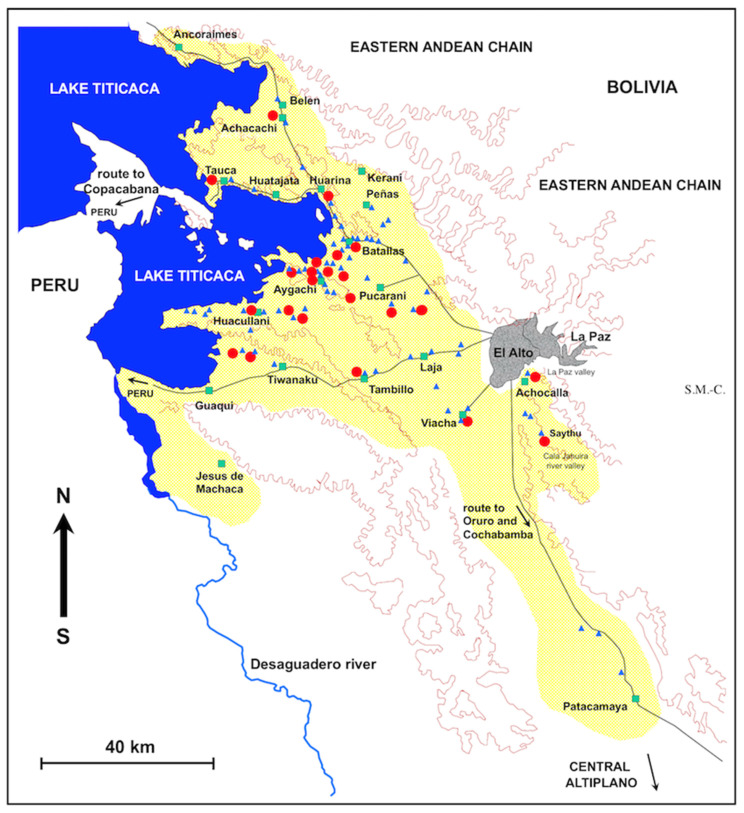
Map of the human fascioliasis hyperendemic area in the Northern Bolivian Altiplano, between Lake Titicaca and the city of El Alto and the valley of La Paz city: Yellow background = total prospected zone; red circles = localities presenting human infection by *Fasciola hepatica*; blue triangles = freshwater collections inhabited by *Galba truncatula* snail vectors; green rectangles = important human localities. Original S. Mas-Coma.

**Figure 2:**
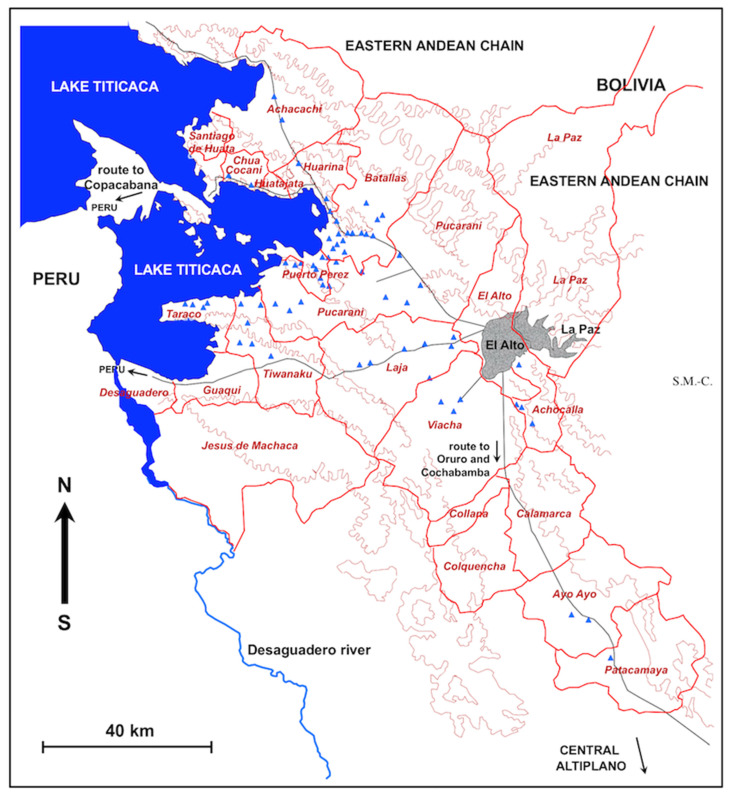
Map of the Northern Bolivian Altiplano showing the distribution of fascioliasis transmission foci according to municipalities affected by the disease and those in the direct neighbourhood. Blue triangles = freshwater collections inhabited by *Galba truncatula* snail vectors; red lines = boundaries of municipalities. Original S. Mas-Coma.

The geographically prospected area in the Northern Bolivian Altiplano, including the location of the surveyed freshwater collections found to harbour lymnaeid snails, i.e., the transmission foci, and human communities, villages, or towns in which human infection was detected, are shown in Figure 1. Municipalities directly involved because they include transmission foci and those affected due to close neighbourhoods are shown in Figure 2. To ascertain the stability of the endemic area, field studies were performed both throughout all seasons and months of the year [51] and along different years of the 1990, 2000, and 2010 decades.

### 2.2. Field Prospections for Transmission Foci

Studies performed throughout the Northern Bolivian Altiplano focused on freshwater collections according to different east–west and north–south transects to cover as much zones as possible inside the aforementioned area. All freshwater collections present along each transect were studied (Figure 1 and Figure 2).

An index card was filed out for each of 67 freshwater collections inhabited by lymnaeid snails, including information on: (i) type of habitat: natural or man-made/modified; (ii) human proximity: close to school or to human community; (iii) freshwater availability: permanent of temporary freshwater collection; (iv) water use: drinking/washing; (v) livestock presence: of cattle, sheep, pigs, and/or donkeys in the surroundings of the freshwater collection; (vi) aquatic and semiaquatic vegetation: mainly concerning the presence of llaytha (*Nostoc*), totora (*Schoenoplectus*), totorillas (*Eleocharis*, *Juncus*, *Lilaea*), berros (*Rorippa*, *Hydrocotyle*, *Mimulus*) and diente de leon (*Taraxacum*). For the assessment of the aquatic and semi-aquatic plant species inhabiting the water collections with lymnaeids, a total of 30 among the aforementioned 67 freshwater collections were analysed both at the end of the raining season and at the end of the dry season.

Only results on the physico-chemical composition of the water, and of air and water temperature in these collections have been published in previous reports [27,51]. To assess the stability of the transmission foci, a total of nine foci were selected for re-visits and re-analyses throughout all seasons and months of the year [51], as well as along different years between 1992 up to 2019 to ascertain potential long-term modifications due to influences of climate change and anthropogenic modifications of the environment [46].

### 2.3. Suspicious Infective Food Habit Assessments

Two localities of the fascioliasis endemic area of the Northern Bolivian Altiplano were selected for surveys on the consumption of freshwater plants by means of a questionnaire:

- Cutusuma, located inside a zone where human infection widely occurs, relatively far from the shore of the Lake Titicaca, along the way from Batallas to Aygachi (Figure 1); a total of 194 persons participated, comprising small children to adult subjects, including mainly school children.
- Tauca, located at the shore of Lake Titicaca, in the road from Huarina to Copacabana (Figure 1), in a zone where human infection shows lower prevalence; a total of 100 persons participated, comprising small children to adult subjects, including mainly school children.

Very young children were helped by their parents to fill out the questionnaire. Taking into account the complexity of the many freshwater plants known to inhabit freshwater habitats in the Bolivian Altiplano [27], the questionnaire was designed to focus on edible plant species by using the locally common Aymara names (and also the respective Spanish translations if needed). The following five common names were included: (i) joskosko (=totorillas); (ii) chullu (=tallo de totora); (iii) sakha (=bulbo de totora); (iv) okororo (=berros); (v) llaytha (=alga parda). Aymara children and adults recognized the aforementioned Aymara names of plants, although in the cases of joskosko and okororo, the Aymara names indeed include more than one plant species and potential confusion may thus not be excluded mainly when dealing with small children. The corresponding Latin systematic names are noted in Table 1.

To analyse the correspondence between consumption of these aquatic and semi-aquatic plant species and the infection by *F. hepatica*, stool samples from all the aforementioned subjects answering the questionnaires were collected together with personal data. To facilitate egg detection, food with a cholecystokinetic (mayonnaise sauce) was given to each participant prior to stool collection. The presence of *F. hepatica* eggs in the faecal samples was qualitatively assessed by direct examination [52], as well as by the techniques of formol-ether concentration [26,53] and the standardized Kato-Katz [54,55].

Additionally, the presence of edible aquatic and semi-aquatic plant species and their frequency was assessed in 30 freshwater collections in which the presence of lymnaeid snails had been previously verified (Table 1). The list of these edible plants was moreover verified to be included in the common knowledge about food traditions of American Indian ethnic groups by literature sources.

### 2.4. Ethnographic Fieldwork Methods Used

The qualitative ethnographic fieldwork method resorting to the participant observation technique was used in many daily visits to the endemic area of the Northern Bolivian Altiplano. Methodological efforts were focused on (i) the participant observation technique, (ii) specific interviews, (iii) information triangulation, (iv) document collection, and (v) data management and analysis, according to standard social research techniques [56]. This field work was repeatedly undertaken during all seasons and throughout a very long period of almost 40 years, namely from 1983 until today, allowing for the assessment of long-term changing trends.

Field analyses were performed in human communities of endemic subzones surrounding transmission foci along the different inter-hilly flat corridors. The main purposes included the obtaining of information and data on crucial aspects related to fascioliasis transmission and the sources for the human infection by *F. hepatica* [13]. Objectives and subzones for this field work were always defined during pre-mission meetings of the participants and health responsible officers in La Paz.

**Figure 3:**
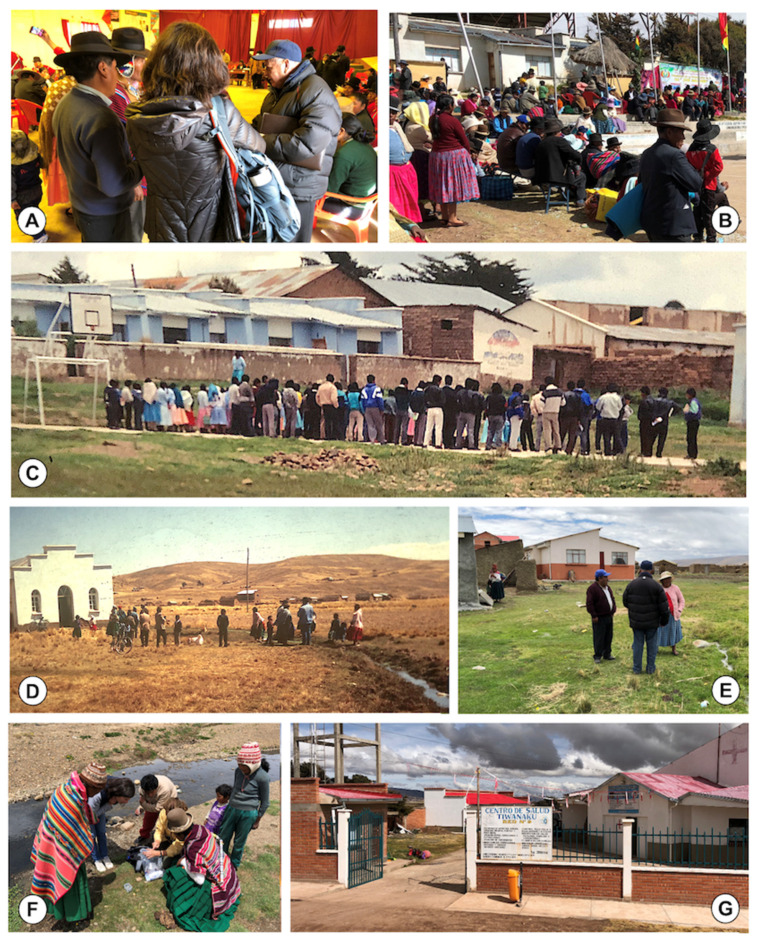
Ethnographic fieldwork: (**A**) Explaining project activities and aims to local Aymara community chiefs (jilakatas and mallkus), in Huacullani; (**B**) Meetings with parents and old people to let know about the disease in Huacullani; (**C**) Giving instructions to children in school, in Huacullani; (**D**) Taking advantage of church visits on Sunday morning for community interviews, in Chojasihui; (**E**) Individual interviews allowed for deeper questioning and fruitful information, in Suriquiña; (**F**) Field teaching of women and children, in charge for livestock grazing, on how to distinguish fascioliasis transmission foci by lymnaeid snail detection, in Challapata; (**G**) Rural health centres in the endemic area were visited for interviews of local physicians and nurses, in Tiwanaku. Photographs S. Mas-Coma.

Activities comprised personal observations by participants, among whom the first and last authors were always involved, with the second author additionally entering when dealing on veterinary aspects. Observations collected by these authors were triangulated with the knowledge of (i) other participants in the same missions, (ii) other local personnel with working experience in the endemic area, such as physicians and nurses working in rural health centres (Figure 3G), (iii) directors and teachers at the rural schools surveyed, (iv) voluntary parents of the children diagnosed, (v) owners of animal reservoirs, and (vi) local Aymara community chiefs (jilakatas and mallkus) (Figure 3A). Both the Spanish and Aymara languages were used. For cross control purposes for this triangulation, mainly spontaneous or semi-structured specifically focused interviews performed in situ were used, although participatory workshops were also organized in the field when appropriate. Aymaras, mainly young and adult women, refuse interviews on aspects such as giving birth, defaecation habits, etc., and adult subjects similarly on other aspects when publicly interviewed. Thus, spontaneous, local, individual normal conversation (Figure 3E) proved to be more fruitful than when dealing with groups of persons where sometimes women exhibit a self-conscious feeling when talking in front of others (Figure 3D).

Previously prepared, colour-printed posters including easy-understandable illustrations adapted to the Altiplanic scenario proved to be highly useful for introductory explanations about the transmission of the disease and human infection sources for both community leaders and parents (Figure 3B), as well as for children (Figure 3C). Interaction with children differed depending on age, by using information sheets differing in contents for the 5–10-year-old and the 11–15-year-old groups. The respective contents were previously agreed with the Bolivian Ministry of Health, La Paz, and specialists of Headquarters of the Pan American Health Organization Headquarters, Washington, DC, USA. Practical training on lymnaeid snail finding and identification of transmission foci was practiced in the field whenever possible (Figure 3F).

To obtain observation samples with representative value, data collections were made in as many human communities close to transmission foci in each endemic zone, corridor, or transect (Table 2) as possible, with the aim to avoid misinterpretations due to disturbing biases in front of potential differences on the aspects analysed, according to local situations. The geographical distribution of the human infection sites analysed by the participant observation technique within the ethnographic fieldwork method covers all zones where transmission foci were found (compare Table 2 and Figure 1). Moreover, these participant observation data obtained in human communities were compared with all information obtained in the different types of transmission foci monthly followed up along a complete one-year period [51].

Participant observation analyses on freshwater collections inhabited by lymnaeid vector snails were performed by the fourth and the last authors; human communities and infection sources mainly concerned the first and last authors. The second author was in charge for the veterinary aspects, and the third author and the last author were involved in data management and analyses. Information on livestock management practices was also obtained by taking advantage of the long-term Programa de Fomento Lechero of the Corporación Regional de Desarrollo de La Paz (CORDEPAZ, El Alto), which covered almost the whole human endemic area throughout the Northern Bolivian Altiplano [58].

Data from the aforementioned activities, and obtained from a total of 23 human localities in which human infection was detected (Table 2), were noted in index cards, protocols, and expedition diary reports. Valuable information on each site was additionally stocked in photograph slides within a large image database. Altogether, this ethnographic fieldwork methodology allowed for a scientifically more accurate descriptive method by means of a quantifying analysis of the different aspects with frequency percentages. These analyses focused on numerous aspects related to fascioliasis transmission and the sources for the human infection by *F. hepatica*, which are here distributed in five categories for an ordered description: (i) household location, (ii) household availabilities, (iii) knowledge about the liver fluke and the disease, (iv) behavioural, traditional, social, and religious aspects, and (v) livestock management.

### 2.5. Literature Search

In the Aymara ethnicity, the knowledge, traditions, and behavioural aspects are orally transmitted from one generation to the subsequent one. Consequently, there is no original Aymara literature available which could be used regarding the objectives of the present study. A few articles published in local journals and books in local editorials, and sometimes also reports within grey literature, sporadically appeared in the last decades. Written information sources appeared before 1995 were already previously reviewed and the extent of local grey literature emphasized [59]. Unfortunately, only a very few deal with the Northern Bolivian Altiplano or neighbouring Aymara zones of interest, and even more rare and pronouncedly scattered were those concerning the aspects linked to the fascioliasis infection sources here in question.

### 2.6. Statistical Analyses

Statistical analyses were carried out using SPSS version 15.0 (SPSS Inc., Chicago, IL, USA). For the evaluation of categorical variables, the chi-squared test or Fisher’s exact test was used. Odds ratio (OR), 95% confidence intervals, and P-values were calculated. Subsequently, stepwise conditional logistic regression was used to determine independent potential risk factors associated with *F. hepatica* egg presence in faeces. Two models (models 1 and 2) were used in the multivariate logistic regression analysis including presence/absence of fascioliasis as dependent variable: (i) model 1 included gender, age, and weight of children as independent variables; (ii) model 2 included gender, age, and weight of children, plus the positive answers to the consumption of the following five raw vegetables (Aymara name/Spanish translation: chullu/tallo de totora, joskosko/totorillas, okororo/berros, sakha/bulbo de totora, and llaytha/alga parda) as independent variables. A P value less than 0.05 was considered statistically significant.

## 3. Results and Discussion

### 3.1. Extent of the Endemic Area and its Strategic Importance

Field prospections were carried out throughout the wide zone of the Northern Bolivian Altiplano eastward from Lake Titicaca and up to the cities of El Alto and La Paz (Figure 1). The endemic area was assessed according to the fascioliasis transmission capacity, i.e., by geographical surveys of all freshwater collections showing appropriate characteristics to enable the existence of lymnaeid snail vectors. A freshwater collection inhabited by lymnaeid snails and presenting known definitive mammal host species in the proximity and surroundings, as mainly sheep and cattle, but also pigs and donkeys, was considered a potential transmission focus. Prospections were performed throughout different seasons and along several years to assure the detection of lymnaeids in each water collection and to establish the stability of the transmission foci. A total of 67 freshwater collections presenting lymnaeid vectors were detected and surveyed (Figure 1, Table 2).

These transmission foci proved to be distributed throughout three main flatlands separated by hilly chains. These inter-hilly flatlands are called corridors and are distinguished according to the main human localities they include (Figure 1):

- The first corridor includes the localities of Pucarani and Batallas, and extends from El Alto in the East up to the shore of the Lake Titicaca in the West, with a northern prolongation through Huarina up to Achacachi and Belen and another westward prolongation along the shore of the Lake up to Tauca. The most recent prospection has demonstrated that lymnaeids have colonized the northward sub-corridor of Peñas and Kerani, where such snails were never found in previous surveys.
- The second corridor extends from Tambillo in the East up to Aygachi, Huacullani and the shore of the Lake Titicaca in the West. The lymnaeids have recently colonized the mountainous hills between Huacullani and the third corridor following the route to Tiwanaku.
- The third corridor concerns the flatland presenting Tiwanaku and Guaqui as main localities and also extends up to the shore of Lake Titicaca in the West.

In the rainy period, superficial water from streams gives rise to floods which spread the snail vectors, increasing plant contamination with metacercariae throughout the plains, as, for instance, in the western part of the Pucarani-Batallas corridor. Such superficial waters run through the three corridors until finally contributing to Lake Titicaca. A first preliminary analysis of the fasciolasis risk distribution throughout these three corridors was assessed by remote sensing mapping by means of the Normalized Difference Vegetation Index (NDVI) [60]. The aforementioned three corridors converge eastward in the large plain of the Kheto river course, along the route from El Alto to Patacamaya, including the locality of Laja and the main town of Viacha. This eastern part of the endemic area includes the valley of Achocalla, besides the La Paz valley, and the Cala Jahuira River valley, as well as the southernmost transmission foci recently colonized by lymnaeids close to Patacamaya (Figure 1).

The whole endemic area thus extends through five provinces of the Departamento de La Paz, namely the province of Omasuyos in the North West, Los Andes in the centre, Murillo in the East, Ingavi in the South West, and the recent southward extension in the province of Aroma [46]. The municipalities presenting infection risk in each one of these five provinces including transmission foci or direct neighbourhood are shown in Figure 2 and listed in Table 2.

This endemic area is of high importance from different points of view, aspects which highlight the national interest in improving the development of the inhabiting Aymara communities:

- It is the main exit for and from the capital La Paz through the recently improved westward road to the city of El Alto.
- The international and national airport is located in El Alto.
- It includes the two terrestrial roads to the Peru border crossings through (i) the northern way of Batallas, Tiquina, and Copacabana, and (i) the southern way of Laja, Guaqui, and Desaguadero.
- It includes the terrestrial roads to Cochabamba, Oruro, Sucre, and other southern parts of the country.
- It includes Lake Titicaca, of undoubted interest from the touristic and naturalistic points of view, including native fauna and flora, landscapes, and opportunities for boating and fishing.
- It includes archeological sites such as the unique ruins of Tiwanaku and the old temple centre of the Aymara empire, of evident touristic, ethnical, and historical attraction, including age-old customs and traditions, folkloric events, etc.

The capacity of fascioliasis to underlie the underdevelopment of the human communities affected should be considered. The devastating effects of a high hyperendemic situation are due to (i) its pathogenicity and morbidity in humans [2,3], (ii) the immune-suppression it induces, and which leads to human coinfections by other pathogenic parasites [41,42], bacteria and viruses [61,62], and (iii) its veterinary impact on livestock, which is crucial for human subsistence at such a high altitude [63]. Therefore, fascioliasis is no doubt the disease of highest negative impact on public health in the Northern Bolivian Altiplano. The need to decrease human infection rates and to control livestock infection by the liver fluke is evident.

### 3.2. Population at Risk, Community Development and Mortality Rates

Estimates of human populations in the municipalities of the endemic area for the years 2012 and 2022 [57], the 67 transmission foci surveyed, and the 23 human localities ethnographically analysed in the present study, together with the respective local prevalences previously found, are given in Table 2. The La Paz Departament is noted to have had 2,767,504 inhabitants in 2012 and to have 3,051,947 inhabitants in 2022, with the great majority distributed in the capital La Paz and the even more populated adjacent city of El Alto.

The rural zone comprising the fascioliasis endemic area included a population at risk of 339,703 inhabitants in 2012 and 329,311 inhabitants in 2022. This timely decrease of about 10,000 inhabitants in the rural zone is due to the migration from rural communities to the urban areas and appears to follow a similar decreasing rate in the provinces of Omasuyos, Los Andes and Ingavi, a little bit slower in Aroma. The fastest-growing city is El Alto, whose immigrants come mainly from the altiplano.

Poverty is a critical social problem in the whole Northern Bolivian Altiplano. It affects mainly the rural populations and their entire social life in general. The available information indicates that, in the Bolivian Altiplano, 99% of the rural inhabitants was living in poverty at the end of the 1990s [64]. Poverty underlies many negative consequences. Families have to devote all their energies to meet their basic needs for food, housing, and clothing, and their resources are too limited to seek an improvement in their living conditions, including their surroundings. Rural illiteracy stands at 26%, higher in women than in men. Extreme poverty and a total lack of opportunity compel the rural populations, especially young people, to migrate to the cities, where they crowd into degraded central districts and slums without basic public services, generally on insalubrious public lands and in hazardous areas, as in El Alto [64].

Facing the extreme poverty and consequent economic, social, and cultural oppression, the affected communities respond with the strategy of a markedly high fecundity. A study performed on women in El Alto immigrants from the rural neighbouring Altiplano reported on females being married very early, when still teenagers, and becoming pregnant many times during their fertile period from 15 to 45 years [65]. Up to 10 children per women and, in given cases, even more, is frequent in the rural zones.

Bolivia has the highest infant mortality rate of whole Latin America. Mortality rates of 12.4% in children aged less than 1 year and 14.7% between 0 and 5 years were reported for the whole country [65,66]. It is easy to conclude that the infant mortality rate should be pronouncedly higher if it is considered that the great majority of the total Bolivian population lives in cities. The 18.4% and 22.3% infant mortality rates estimated for the Bolivian Altiplano at the end of last century [64,67] may thus also be underestimations.

The majority of deaths occur within the age range of 0–14 years. The high mortality rate in infancy leads the parents to not declare the newborn until the age of 2 years [65], even pronouncedly later in the rural areas, up to before schooling when reaching 4–5 years of age. Therefore, official rates of mortality based on registered data underestimate the real infant mortality rate in the Aymara rural area.

Malnutrition in a very high altitude inhospitable environment is the most frequently evoked explanation for this high infant mortality rate, as well as for the percentage of undersized and underweight children, in the relatively scarce literature analysing this situation [68,69]. Surprisingly, only a few articles and books consider the potential importance of infectious disease as the main cause or one of the causes [64,67,69,70]. However, early in the 1990s, there were observations which confirmed a higher morbidity and mortality from respiratory, gallbladder, and liver disease, reported in a study highlighting the paucity of information on disease and disability in the typical highland Aymara communities [71]. Diarrhea was reported as one of the predominant causes of the high infant mortality [65].

Nonetheless, fascioliasis is known to be the most frequent parasitic disease in the inhabitants of the Northern Bolivian Altiplano since the 1980s, as shown by a wide review of grey literature found in La Paz [59]. The first case in Bolivia was reported in March 1939 [72], another 20 additional cases were subsequently reported from a hospital of La Paz in the same year [73], and an ectopic cutaneous case and a biliary case surgically solved in the Hospital General of La Paz were reported many years later, in 1962 [74]. The veterinary impact of fascioliasis on the livestock of the Northern Bolivian Altiplano was already detected in 1973, with the description of sheep mortality caused by acute fascioliasis [75]. The mortality rate by *Fasciola* in sheep was estimated to be not less than 15-25% annually. Outbreaks of acute fascioliasis were recorded from the end of April to the beginning of August, especially from May to July, or in the dry season of June and July [76]. It should be considered that sheep is the most numerous livestock species and that it has recently proven, both experimentally and in the field, to be the main reservoir species in this Altiplanic hyperendemic area [47].

A study in the official slaughterhouse of La Paz showed a fascioliasis prevalence at similar annual rates in cattle during the 1975–1979 period, already indicating the disease to be endemic. In humans, an additional follow up analysis of the patients diagnosed in several hospitals of La Paz compiled a total of 95 cases along a 15 year period from 1970 to 1985, highlighting possible repercussions in malnutrition and suggesting that the fascioliasis endemic of the Altiplano had an urban extension in the capital [77]. Transmission foci have, however, never been found in the valley of La Paz so far, not even in our surveys carried out southward from the Cala Coto, at altitudes lower than 3200 m a.s.l. The only exception appears to be the high subvalley of Achocalla, besides El Alto and at altitudes of 3750–3850 m a.s.l. (Figure 1 and Figure 2). Consequently, fascioliasis patients diagnosed in La Paz hospitals should have been infected in the rural Altiplano or in uncontrolled city markets [78] or ambulant hawking by “cholitas” in city streets selling plants and fruits from the endemic rural neigbouring areas, as well as beverages made with such vegetables [13].

Child mortality caused by liver fluke infection was repeatedly reported in the second half of the 1980s. A 10-year-old child died despite being treated in the Hospital del Niño of La Paz in 1986 [79]. Two other children also died in the Hospital Juan XXIII of La Paz in the same year [80]. Up to 12 fatal cases among tens of severe cases and hundreds with moderate disease were reported from the Altiplanic community of Corapata [81]. In Cullucachi, another community in the Altiplano, 15 deaths among 20 newborns due to unidentified causes, and 8 deaths among children less than 5 years old because of gastrointestinal problems were reported during only one year period [82]. The alarm caused by these emerging data led the authorities to react. Thus, fascioliasis was listed as the first of the three most important zoonoses (1.—Fascioliasis; 2.—Teniasis/Cisticercosis; 3.—Hidatidosis). The importance of these zoonoses led the Bolivian Health Ministry to create the «Comité Técnico de Vigilancia y Control de Zoonosis» in 1989, to coordinate policies, strategies, programmes, and activities of public and private organizations, in the fight against these parasitic diseases. A Ministerial Resolution was therefore published on 4th August 1989 by the Ministerio de Previsión Social y Salud Pública and Ministerio de Asuntos Campesinos y Agropecuarios, La Paz [59,83]. The first mission to La Paz and the Altiplano endemic area by members of the Valencia WHO Collaborating Centre was in March 1992 and the collaboration of WHO regarding fascioliasis has been kept uninterruptedly since then up to the present.

It has been recently demonstrated that child infection by the liver fluke may occur very early in life, i.e., including very young children aged only a few months [84]. Although in the Northern Bolivian Altiplano there is only a single report of a preschool child infected, namely a 2-year-old girl [26], it is logical to suspect that this is due to the fact that surveys on infancy have been always performed on schoolchildren, including from the age of 5 years [27,59]. Very early infection in preschool children may thus also be expected to happen in the Bolivian Altiplano, taking into account the high infection risk throughout the hyperendemic area and the food and water drinking habits of the Aymara inhabiting these very high altitude environments. The availability of the new preservative/diluent CoproGuard, developed for the preservation of liver fluke coproantigens [85], may be useful for rural mothers to collect stool samples from their small children and to carry them to La Paz where a coproantigen test may be applied for fascioliasis diagnosis. Indeed, the usefulness of a coproantigen test has already been successfully evaluated in the Altiplano [44].

Precocious infections in preschool children of less than 5 years and in very young school children of less than 10 years indicates that such infected children reach the chronic and advanced chronic phases of the disease when they are still very young. They are thus affected by the immunosuppression induced by the liver fluke during the chronic stage, which leads to an increase in their susceptibility to become co-infected by other pathogenic parasites and microorganisms as bacteria and viruses [14,61,62]. Co-infections with other protozoans, including associations with pathogens such as *Giardia intestinalis*, *Balantidium coli*, and *Cryptosporidium* spp., and with helminths as *Ascaris lumbricoides* and *Trichuris trichiura*, among many others, have been reported in the school children of the Northern Bolivian Altiplano [41,42]. Up to a maximum of eight different parasite species have been found in *F. hepatica*-infected children in this hyperendemic area, among which were up to four other parasites of recognized pathogenicity [41]. These co-infections are individual situations of high morbidity which may no doubt also underlie the high infant mortality [86]. Additionally, morbidity should increase by accumulation of liver flukes as a consequence of reinfections caused by the high transmission risk and the absence of premunition in fascioliasis [38,39]. The higher pathogenicity of high liver fluke intensities is well known and the high epg rates reported in Altiplanic children lead to suspect such reinfections to be frequent in this hyperendemic area.

### 3.3. Characteristics of the Transmission Foci

The geographical distribution of the freshwater collections harbouring lymnaeid snails is shown in Figure 1. Despite slight differences of the morphology of the shell, which had previously led to believe that two different American lymnaeid species were involved, *L. viatrix* and *L. cubensis* [76], subsequent phenotypic analyses demonstrated that it was indeed nothing more than a wide intraspecific variability of the populations and that only one lymnaeid species was involved, namely *G. truncatula* [87]. Ribosomal DNA ITS-2 and ITS-1 sequencing allowed us to later verify this specific ascription and to ascertain their introduction from Europe during the colonization period [35]. Additional sequencing of the complete length of the two mitochondrial DNA genes *cox*1 and *nad*1 has more recently proven the monomorphic characteristics of all *G. truncatula* populations throughout the Northern Bolivian Altiplano. This clonicity suggests that all present populations should have derived from an initial unique founder specimen by selfing reproduction and hence a similar very high transmission capacity by all local populations [46].

The spatial distribution of the transmission foci appears to be patchy [27,46], as is typical in other freshwater snail transmitted diseases, as it is in the case of schistosomiasis [88]. Recently, a spread of the external outline of the distribution of the transmission area has been observed. Lymnaeids have been found in three places where they were not present before, namely (i) northward in the Peñas subcorridor, (iI) in the hills between the corridors of Huacullani and Tiwanaku, and (iii) southward in the Patacamaya zone [46]. All these new findings are in places of altitudes higher than those of the previously detected foci [27]. These discoveries indicate that the distribution of the transmission foci is temporarily dynamic and suggests the need to periodically perform field surveys to assess the external extent of the endemic area, in the way to include the new transmission zones into the control activities. The new Patacamaya zone implies the need to include the province of Aroma in the control initiatives for the first time. A study presently underway has the purpose to verify whether this spread involving higher altitudes [46] is related to climate change.

The distribution of the transmission foci inside the past established limits of the endemic area appears to be appropriately stable along different years. This confirms a situation of endemicity, which explains the prevalence results obtained in surveys of both humans and livestock along many years. Consequently, terms as outbreak or epidemics [89] should be considered misinterpretations of the transmission and epidemiological situation. Only human action appears to modify given transmission foci, as for instance along the construction of paved roads, implementation of irrigations, and improvement of water sources [48].

A detailed analysis of the spatial distribution of the lymnaeid-inhabited freshwater collections clearly shows a concentration of the transmission foci in the proximity of the Lake Titicaca, so that the municipalities reaching the shore of the Lake are the most affected (Figure 1 and Figure 2). Intermediate zones at already some distance from the Lake appear intermediate, with a lower number of transmission foci, such as the municipality of Laja and parts of the municipalities of Batallas, Pucarani, or Tiwanaku. An opposite situation is that of the municipalities further away from the Lake, where foci are scarce, as in Viacha, Achocalla, Ayo Ayo, and Patacamaya. Three facts explain this gradual distribution:

- The decreasing slope from the Eastern Andean Chain (with altitudes up to more than 6000 m) down to the Lake Titicaca (at 3820 m a.s.l.), as indeed all freshwater collections in the endemic area, whether superficial or phreatic, giving rise to efflorescence, come from this chain;
- The distance from the Eastern Andean Chain explains the gradual disappearance of transmission foci when far away, such as in the southern part of the Tiwanaku corridor; the long Kheto river allows for the southward spread up to Patacamaya;
- The progressively decreasing night temperature when increasing the distance from the Lake, because of the loss of the mildering climatic effect of the Lake [90,91].

The concentrated presence of transmission foci close to the eastern shore of the small southern lake of the Titicaca (Lago Menor) allows us to understand the traditional belief of the local Aymara inhabitants about a link of “talpalako” with the Lake. Indeed, the salinity of the Lake Titicaca, of 1343–1521 µScm in the Small Lake depending on the season [92], does not allow the survival of *G. truncatula* in its waters. Salinity has adverse effects on lymnaeid vector species, and studies on the malacofauna of Lake Titicaca have never found lymnaeids in its waters [93]. Therefore, the Lake in fact plays the role of a westward barrier for the disease. Very wide surveys of up to 30 collections, at the end of the rainy season and the end of the dry season, allowed for the physico-chemical characterization of their waters [27], but could not find any explanation about why given “bofedales” are never colonized by lymnaeids, such as those in the Batallas municipality zone southward of the Peñas subcorridor. Large superficial extents of salt are found in the zones between Belen and Ancoraimes in the north, and also in the Capiri zone, southward from Viacha, explaining why lymnaeids are absent in the water collections on these zones [27].

**Figure 4:**
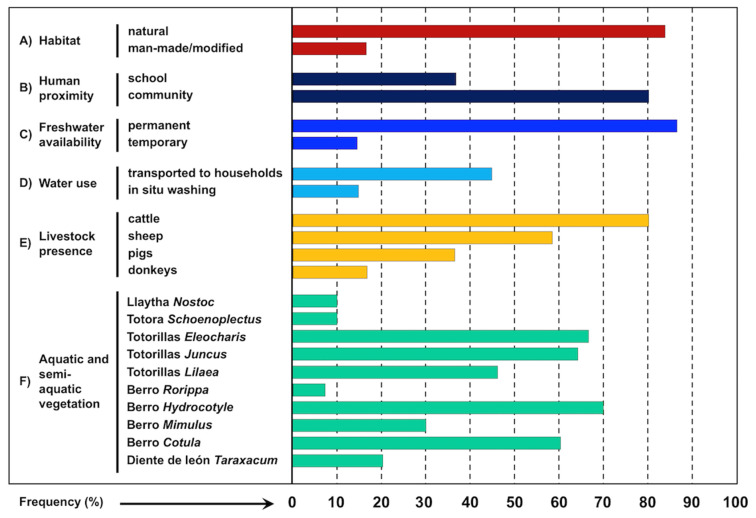
Results (%) of the quantitative analyses of the characteristics of the 67 freshwater collections inhabited by *Galba truncatula* snail vectors surveyed throughout the human fascioliasis hyperendemic area in the Northern Bolivian Altiplano, including frequency percentages of presence of main infection-linked vegetables in a total of 30 of these fascioliasis transmission foci which were analysed both at the end of the raining season and at the end of the dry season of different years.

Results of the studies of the 67 lymnaeid-inhabited freshwater collections prospected throughout the endemic area and including quantified aspects are shown in Figure 4. Most of the transmission foci are natural freshwater collections, including from rivers to small streams, derived flooded areas, or natural spring pools whose waters are provided from quite superficial phreatic layers leading the underground waters from the Eastern Andean Chain [51]. Man-made habitats are less frequent lymnaeid-inhabited freshwater collections, including mainly small canals, artificial canalizations, or flooded surroundings of wells or broken fountains (Figure 4A). Moreover, most of the lymnaeid-inhabited freshwater collections are permanent habitats in which lymnaeids are present throughout the year, and only a few may be considered temporary (Figure 4C), because of the absence of superficial water during a very few months along which the lymnaeids survive buried into the humid soil [51]. The high evapotranspiration rates at such a very high altitude do not allow water from rainfall to stay sufficient time as to be colonized by lymnaeids [32]. A detailed analysis of the monthly dynamics of the Altiplanic *G. truncatula* populations has recently demonstrated that Altiplanic lymnaeids follow a two generations/year pattern in permanent water habitats, and a one generation/year pattern in habitats drying out for months, and that control measures can be extended from one part of the endemic area to another despite local dynamic differences, i.e., nothing suggests different responses to the local application of similar control measures [51].

The proximity of the transmission foci to schools and human communities and villages should be highlighted (Figure 4B). Indeed, several of the lymnaeid-inhabited freshwater collections surveyed were used for water collection and transported to the household for food preparation, washing, or drinking, although washing of clothes is also performed at rivers (Figure 4D).

Registered observations showed that livestock is usually present in the close surroundings of lymnaeid-inhabited freshwater collections, mainly cattle and sheep, less frequently a few pigs, and sometimes also one or two donkeys (Figure 4E).

The surveys of these freshwater collections inhabited by lymnaeids allowed for the quantification of the frequency of plants noted by the Aymara inhabitants to be included in their diet or usually chewed and/or sucked by children when in the field (Figure 4F). The most frequent may be included in the local terms of “totorillas” and “berros”, both of which indeed include different plant species (Figure 4F). Local Aymara and Spanish names, together with scientific species names, and quantification of their frequency are noted in Table 1. The low frequency of “llaytha” should be emphasized, given that it is one of the most mentioned by the Aymara inhabitants. The “totora” is also repeatedly referred to, but a negative association was observed between this plant and lymnaeid presence, most probably because of the noxious secretions of its roots [94,95]. Lymnaeids could be rarely found in a water collection in which totoras were present, although they were always far away from this plant.

### 3.4. Food Infection Sources

In the locality of Cutusuma, from the 194 participants (100 males and 94 females; 135 children and 59 adults) answering the questionnaires, only 128 subjects furnished a stool sample whose coprological analysis allowed for the detection of liver fluke eggs in 42 individuals (prevalence = 32.8%), among whom 20 from 61 males (32.7%) and 22 from 67 females (32.8%). The distribution of the infected subjects according to age groups was the following: 1–10 years: 26.2%; 11–20 years: 17.2%; 21–30 years: 4%; 31–40 years: 2.7%; 41–50 years: 1.8%; 51–60 years: 0.4%; >60 years: 1%.

In Tauca, from the 100 participants (52 males and 48 females; 72 children and 28 adults) answering the questionnaires, only 38 subjects furnished a stool sample whose coprological analysis allowed for the detection of liver fluke eggs in 8 individuals (prevalence = 21.0%), among whom 4 from 20 males (20.0%) and 4 from 18 females (22.2%). The distribution of the infected subjects according to age groups was the following: 1–10 years: 0%; 11–20 years: 2.3%; 21–30 years: 0%; 31–40 years: 0.4%; 41–50 years: 0.2%; 51–60 years: 0%; >60 years: 0%.

The aforementioned data allowed for the analysis of the distribution of the positive answers in the questionnaires regarding plant consumption in infected subjects of Cutusuma, but unfortunately not in Tauca because the infection rate was too low to obtain significant results in this second locality.

The analysis of the consumption of aquatic plants linked by Aymara inhabitants to human infection by the liver fluke, demonstrates that all the five plants in question are usually consumed in both localities (Table 3 and Table 4). Differences appear when comparing between the two localities, which may probably be related to their location, i.e., far from the Lake Titicaca (Cutusuma) and at the shore of the Lake (Tauca).

**Figure 5:**
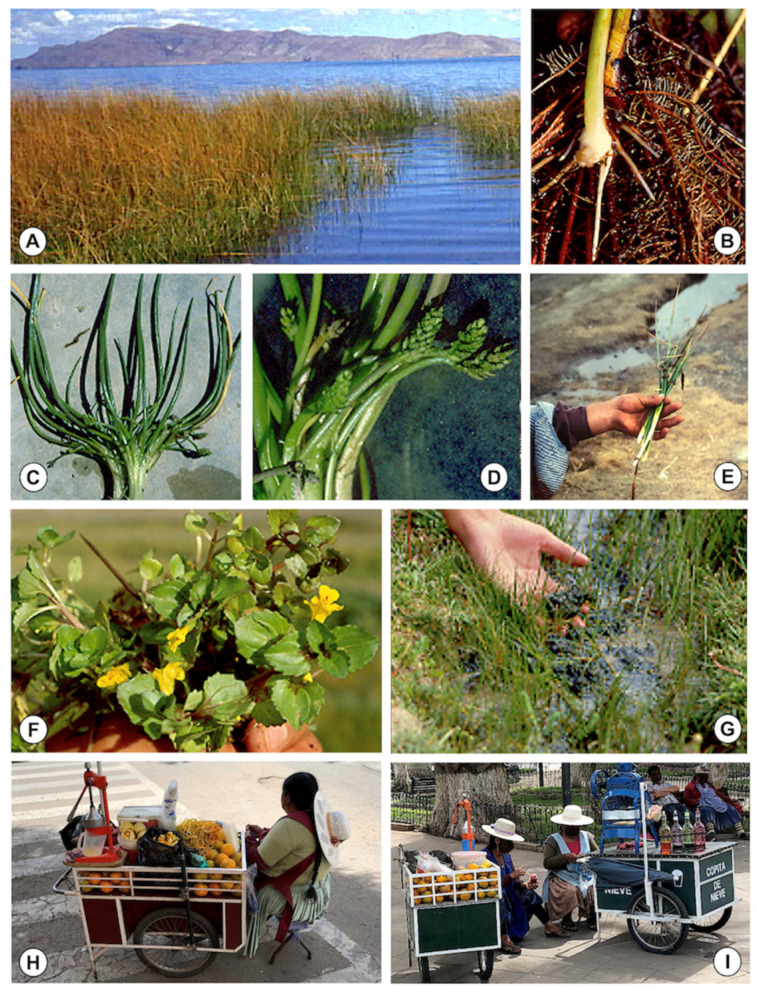
Consumption of potentially transmitting local plants: (**A**,**B**) Stems of totora (*Schoenoplectus totora*) at the shore of Lake Titicaca (**A**) and detail of its bulb (**B**); (**C**–**E**) The local word “totorillla” concerns different freshwater plant species such as *Lilaea* sp. (**C**,**D**), *Eleocharis* spp. (**E**) and others such as *Juncus* spp. (**F**) The word “okororo” is used by Aymaras to refer to several similar freshwater plants as *Mimulus glabratus*; (**G**) Llaytha is a biomass of colonies of a *Nostoc* cyanobacterium which grows in wet places and known as “cochayuyo” by the Aymara inhabitants; (**H**,**I**) Ambulant hawking by “cholitas” in city streets implies selling of plants and fruits from the endemic rural neigbouring areas, as well as beverages made with such vegetables, which potentially underly urban infection risk. (**A**,**E**,**H**,**I**) Photographs S. Mas-Coma; (**B**–**D**,**F**,**G**) Photographs R. Angles.

The analysis according to gender is included in Table 3. When considering all the participants answering the questionnaires, significant differences between males and females appear in the consumption, both in Cutusuma for chullu (Figure 5A) and joskosko (Figure 5C–E) and in Tauca for okororo (Figure 5F). In all these plants, the consumption by females is higher than by males. In another study in the locality of Calasaya, relatively close to Cutusuma, women appeared more likely than males to have fascioliasis, with a prevalence of 38% vs. 20% [89]. Later, no significant difference in prevalence between males and females was found in the largest survey covering the whole endemic area, but infection intensity still proved to be significantly higher in females [26]. These higher burdens in girls and women suggest reinfections to be more frequent in females than in males [38,39], similarly as observed in the human fascioliasis hyperendemic area of the Nile Delta in Egypt [96]. Indeed, many of these high burden cases may be the consequence of repeated and accumulative reinfections, because of the absence of premunition in fascioliasis [14] and differences of behaviour linked to gender [97].

Interestingly, when analysing the gender only inside the infected subjects, berros (Figure 5F) appear to be significantly and pronouncedly more consumed by males than by females in Cutusuma.

Results obtained when comparing age groups, i.e., children versus adult subjects, are noted in Table 4. Significant differences appear in the analyses of okororo (Figure 5F), sakha (Figure 5B) and llaytha (Figure 5G) in Cutusuma and only of okororo in Tauca when considering all participants. In all these plants, the numbers of positive answers in adult subjects were pronouncedly higher than in children, which indicates that the consumption of these risky plants increase with age. No significant difference appeared when considering only infected subjects in Cutusuma.

A multivariate logistic regression analysis of the different data obtained from children of the locality of Cutusuma, by using two models which include the presence/absence of fascioliasis infection as a dependent variable, is included in Table 5. Age and weight appear with a significantly higher liver fluke infection risk in the two models. Interestingly, when including the consumed vegetables in model 2, the age coefficient increases, suggesting that these vegetables play a role in liver fluke infection risk in the Northern Bolivian Altiplano.

Although none of the five vegetables reach significance, both the totorillas or joskosko (Figure 5C–E) and the alga parda or llaytha (Figure 5G) show coefficients markedly higher than 1, which could be interpreted as more risky. Indeed, children usually enjoy sucking and/or chewing the juicy white initial part of the stem of plants such as totorilllas. In an analysis of different aquatic and semiaquatic plants in a fascioliasis focus of the Northern Bolivian Altiplano, the totorilla *Eleocharis* sp. (Figure 5E) was experimentally proved to harbour the highest number of metacercariae per 100 g of plant (50.9), only surpassed by Compositae plant species (56.3), whereas all other plant species assayed showed pronouncedly lower rates [76]. The daily journey from home to school and back [13], as well as livestock herding by women and children (see below), offer repeated opportunities for collecting and putting totorillas into the mouth.

The involvement of Compositae plant species for metacercariae fixation [76] should also be taken into account, because of their high frequency in fascioliasis freshwater foci in the Northern Bolivian Altiplano (Table 1). Not only “berro”-like vegetables such as *Cotula mexicana* but mainly dandelion leaves (*Taraxacum* spp., known as qhanapaku in Aymara language) have been reported to have a role in human infection in France and Argentina [13,20,98].

Llaytha or cochayuyo are the names given to blue-green cyanobacterium microalga macrocolonies of the species *Nostoc sphaericum* (Cyanophyta: Nostocales) which are present in 10% of the freshwater collections inhabited by lymnaeid snails in the Northern Bolivian Altiplano (Table 1). The colonies of this cyanobacterium give rise to a biomass (Figure 5G) which grows in the Andean highland wetlands. It is collected, sun-dried, and used for human consumption in the diet of rural Andean communities, according to an ancestral tradition dating from pre-Columbian times and transmitted through generations [99]. Once dried, it may also be sold and afterwards re-hydrated in restaurants where it is used as spicy condiment. Unfortunately, sun-drying may not be sufficient to avoid liver fluke infection risk, because metacercariae are very resistant [13] and may keep their viability and infectivity if the biomass is kept dried for a short time of only months [33]. *Nostoc* cyanobacteriae are known to be also present in the Peruvian valleys [100], where freshwater lymnaeid snails are also giving rise to human fascioliasis hyperendemic areas such as in the Mantaro-Junin valley [101,102] and the Cajamarca province [103,104].

Although all of the aforementioned analyses concern the rural endemic area, uncontrolled ambulant selling of beverages made from vegetables, and also the vegetables themselves, collected from the field allow for the spread of the liver fluke infection risk to urban areas, including mainly the city of La Paz where such type of commerce carried out by the so-called “cholitas” is very frequent almost everywhere (Figure 5H,I), but also in El Alto. The immediate neighbourhood of the endemic area to these two cities facilitates such a type of activities. Local beverages and juices made from vegetables are known to be involved in human infection. Contamination of such beverages and juices may be from either the plants or the natural water used for their washing and beverage production [13]. “Emolientes” (emollients) are warm aqueous drinks made from various medicinal plants, mainly alfalfa and watercress. Such warm beverages, supposedly appropriate for liver diseases among other illnesses, rise as a risk factor in questionnaire surveys in endemic areas [101,105,106] and also in the anamnesis of infected patients in hospitals [107]. Specifically, the juice of alfalfa (*Medicago sativa*), usually cultivated as livestock fodder, repeatedly appears to be correlated with liver fluke infection, whether in Mexico [17] or rural Andean endemic areas of Peru [106]. Alfalfa is used in traditional medicine because of its high content in amino acids, proteins, enzymes, vitamins, and calcium [108].

### 3.5. Water Infection Sources

Emphasis has been given to natural freshwater as fascioliasis infection source in a recent worldwide review, increasing studies indicating it to be of pronouncedly more importance for human infection than previously considered [13].

**Figure 6:**
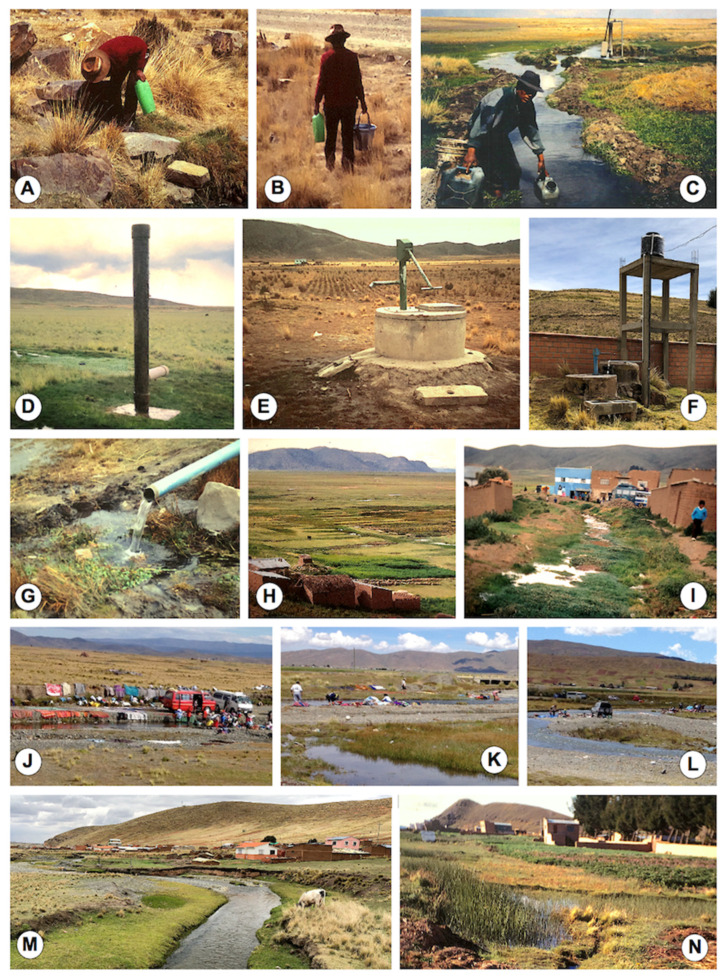
Natural water as fascioliasis infection source: (**A**–**C**) Freshwater is collected in the field to take home for food preparation, drinking and washing of kitchen utensils and clothes, sporadically for personal hygiene; (**D**,**E**) Lymnaeid snails usually colonize the flooded ground surrounding fountains as in Laja (**D**) and wells as in Chijipata Alto (**E**); (**F**) Motorized water availability is being recently installed in rural schools, as in Santa Rosa; (**G**) Water canalizations used for irrigation giving rise to habitats for lymnaeid vectors, in Chijipata Alto; (**H**) Reduced crops are only for local supply, in Cutusuma; (**I**) Floating metacercariae implying infection risk by water drinking were found in the small river inside the village of Tambillo; (**J**,**K**) Washing clothes is traditionally practiced in rivers inhabited by lymnaeid vectors, in Rio Karawisa: (**L**) Recreational activities are usually performed at wide lymnaeid-inhabited rivers by families with small children in weekends, in Batallas; (**M**,**N**) Closeness of freshwater collections is crucial in the decision of where to construct a dwelling, in San Calixto (**M**) and Copancara (**N**). (**A**,**B**,**D**–**N**) Photographs S. Mas-Coma; (**C**) Photograph J.G. Esteban. C modified from Mas-Coma et al., Parasitology, 2018, 145, 1665–1699.

The Aymara culture worships “mother nature” (“Pachamama”) and claims that water, as an element of nature involved in the daily feeding and drinking of the human community and in breeding plants and animals, should come from the springs where life flows and water clarity manifests its purity and living essence, and that dead (boiled) or contaminated water should be avoided [109]. In the rural communities of the Altiplano, Aymara families use at-home natural water collected from freshwater collections (Figure 6A–C) located close to the household for food preparation, washing of kitchen utensils and clothes, and also drinking, and rarely for their personal hygiene. Moreover, children drink water from field collections along their daily walk from home to school and back, and similarly do women and children when in charge for livestock herding.

In the surveys of the transmission foci, the occasion arrived to see male inhabitants collecting waters from freshwater collections which proved to harbour lymnaeid populations, such as water spring pools (Figure 6A,B) and also floods from broken fountains (Figure 6C,D). These freshwater collections offered a source of water shared by humans and livestock, and therefore contamination by faecal infectious agents was assured. The high infection rates of the Altiplanic inhabitants by parasites of hydric transmission, such as several species of amoebae, *Giardia intestinalis* [41,42], *Cryptosporidium* spp. [110] and *Blastocystis hominis* [41,42,111], are indicators of the frequent human infection through water.

The potential relationship between liver fluke infection and the consumption of contaminated water from natural freshwater collections playing a shared source for humans and animals in the Altiplanic locality of Corapata was suggested early on [81].

The overall local tradition to use water from such natural origins, not only for drinking but also for the washing of field-collected vegetables, was also highlighted in Cullucachi [82]. Drinking of contaminated natural water was also associated with growth retardation of Aymara children [67], with gastroenteritis, enteritis, and other diarrheic diseases throughout the Altiplano [64], and with a higher risk of infection by intestinal pathogens in Taraco [70].

Protected wells began to be constructed in the zones neighbouring the Lake Titicaca in the second half of the 1980s. Lymnaeids are usually found in their surrounding floods (Figure 6E). These are shallow wells, no more than 10 m deep, although deep wells of less than 110 m are found far from the Lake, as in El Alto [64]. Unfortunately, people do not frequently visit these wells for obtaining water for domestic consumption, despite its purpose. A total of 50% of the Aymara families were estimated to not use them [67]. Motorized systems of water supply are recently being implemented, however, in the yard of primary schools located just next to transmission foci (Figure 6F).

Uncontrolled artificial tube canalizations providing a permanent flow of water give rise to lymnaeid inhabited floods (Figure 6G), similarly as man-made canals for the irrigation of small croplands of vegetable cultivation for consumption of the local community (Figure 6H). The fascioliasis infection risk was even proved in streams crossing small villages, when liver fluke metacercariae were found in the waters just in the centre of such a community (Figure 6I) [112].

Large rivers coming from snow melting in the high mountains of the Eastern Andean Chain are used by communities for the washing of all kinds of clothes (Figure 6J) and also individual families (Figure 6K). Lymnaeids are found in large numbers in the banks of such rivers, as the Karawisa river. These washing activities have already been seen to be infection risky in other endemic areas [17]. Such river zones are also used by families from the close cities of La Paz and El Alto for recreational activities and for the games of their children during the weekends (Figure 6L). The infection risk of these kinds of recreational activities is already known in other neighbouring South American countries [20].

The high risk of human infection is also highlighted by the proximity of different types of lymnaeid-inhabited freshwater collections to communities and dwellings throughout the whole Northern Bolivian Altiplano hyperendemic area (Figure 6M,N). The presence of livestock in the surroundings of the households where these water collections are present assures their potential role in disease transmission.

### 3.6. Household Characteristics

**Figure 7:**
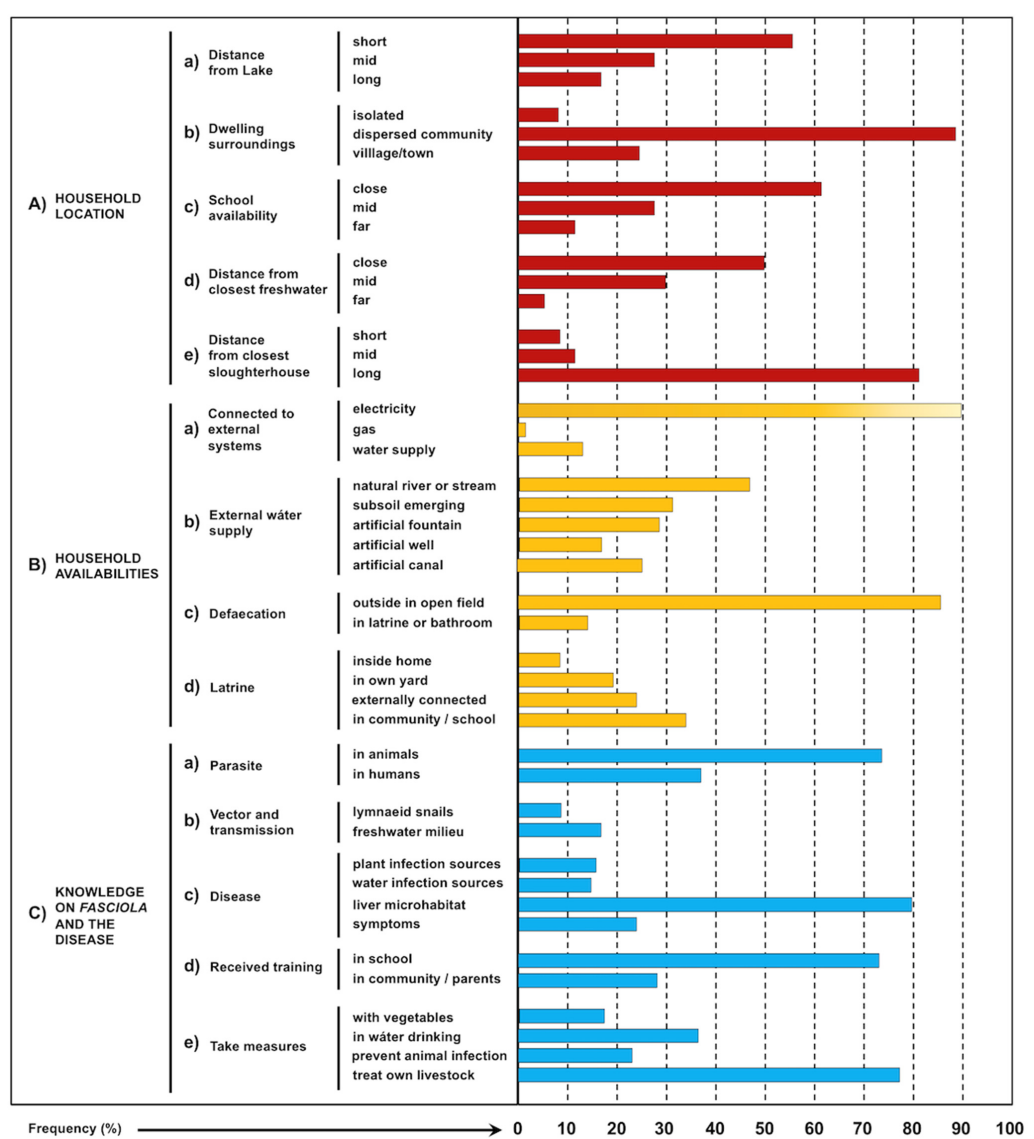
Results (%) of the quantitative analyses of the characteristics of 23 Aymara communities presenting human infection by *Fasciola hepatica*, covering all zones of the human fascioliasis hyperendemic area in the Northern Bolivian Altiplano with transmission foci in each endemic inter-hilly flat corridor or transect, including information obtained in the ethnographic fieldwork, during all seasons and throughout a very long period of 35 years, about aspects linked to fascioliasis transmission and human infection: (**A**) household location; (**B**) household availabilities (degraded colour in “access to electricity” refers to pronounced improvement along the last three decades up to the present 90%); (**C**) knowledge of the inhabitants on *Fasciola*, the infection by this parasite, and the disease it causes.

**Figure 8:**
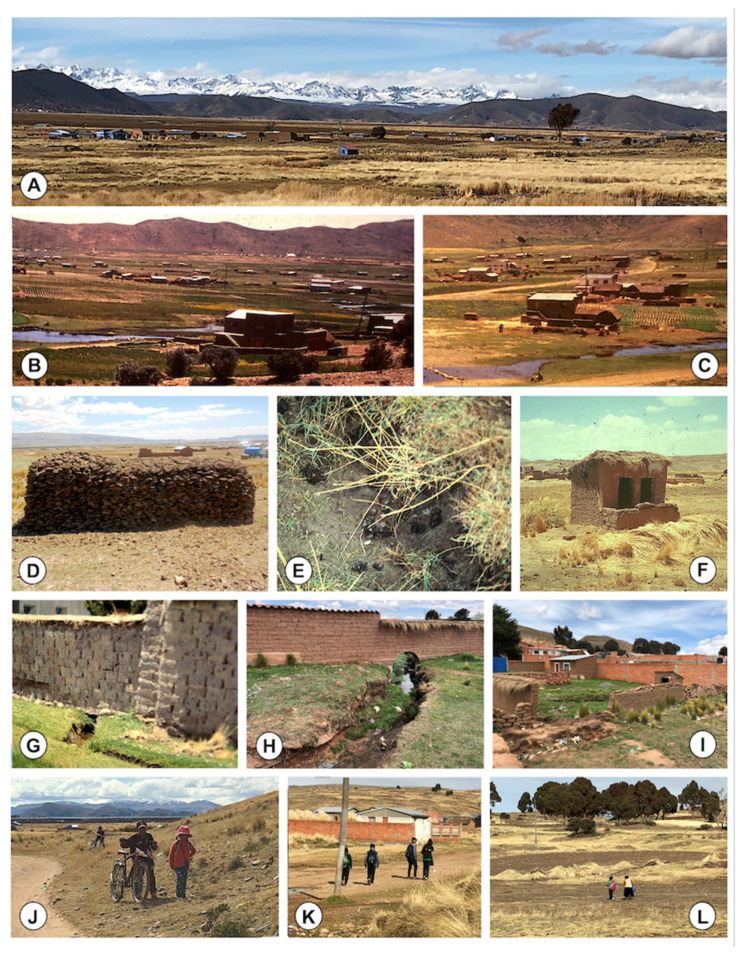
Characteristics of the households: (**A**–**C**) Aymara communities use to live in dispersed dwellings, with livestock freely running and lymnaeid-inhabited freshwater collections in between and alongside, as in Huacullani (**A**) and Cutusuma (**B**,**C**); (**D**) heaped dried cattle manure, in Ticuyo; (**E**) Outside defaecation is traditionally practiced in places where freshwater is present; (**F**) Latrines for communities are scarcely found in the open field, as in Chiripujo; (**G**–**I**) Waste canalization coming from the latrine (**I**), crossing the outdoor household walls, and externally contacting lymnaeid-inhabited freshwater collections (**G**,**H**) are sometimes found, as in Chirapaca (**G**) and Peñas (**H**,**I**); (**J**–**L**) Children daily walk from home to school and back through the field, as in Santa Rosa (**J**) and Huacullani (**K**,**L**). (**A**–**C**, **E**–**L**) Photographs S. Mas-Coma; (**D**) Photograph R. Angles.

Quantifications of the data on household characteristics of importance regarding fascioliasis transmission and risk of human infection by *F. hepatica*, obtained in the ethnographic studies by author observations and personal interviews in the surveys of the localities in which human infection was diagnosed, are shown in Figure 7, and illustrating photographs of crucial aspects are included in Figure 8.

Most of the localities with human infection are located close to the eastern shore of Lake Titicaca, and there is a gradual decrease in the number of the infected localities with the increasing distance from the Lake (Figure 1, Figure 2 and Figure 7(Aa)). This agrees with the same distributional pattern shown by the transmission foci. Indeed, a link of the human prevalences with the proximity of the lymnaeid-inhabited freshwater collections to the localities was already highlighted [27].

In the rural zones where more transmission foci are present, households are distributed mainly in dispersed communities (Figure 8A–C), with freshwater collections close or even in between the dwellings. Distances separating the dwellings are sufficient as to allow for livestock freely running in between, thus assuring faecal contamination of the local water collections. More isolated households are rare, and dwellings arranged in an organized manner around streets are only found in the most populated villages or towns, such as Viacha and Batallas (Figure 7(Ab)).

Although numerous schools have been implemented throughout the whole fascioliasis endemic area of the Northern Altiplano, differences exist regarding the distance of the household and the school (Figure 7(Ac)). The importance of this distance relies on the daily walks children have to and from the school (Figure 8J–L).

The increase in prevalence in children from 5 years onwards is generally attributed to the high infection risk along such walking trips twice a day, because of chewing and/or sucking tasty freshwater vegetables or drinking from natural water collections [13], mainly in the age groups up to 11 years after which prevalence begin to decrease [26].

In both the dispersed household communities and isolated dwellings, the proximity of a water collection is considered fundamental in the moment of deciding where to construct. Thus, a relatively high proportion of households has freshwater collections in very close or mid proximity, and only a very few are located far from the closest water availability (Figure 7(Ad)).

The main closest slaughterhouse is located in El Alto, which is too far for most of the endemic area (Figure 7(Ae)). A small slaughterhouse was built up besides Batallas at the beginning of the 1990s, but the requirement to pay for its use imposed by the local authorities rapidly led to its obsolescence and its present abandonment. Thus, uncontrolled sheep and pig slaughtering occurs once per week at the river of Batallas (usually sheep on Saturdays and pigs on Thursdays), where liver fluke infected livers are usually found by the families killing their animals [48]. Repercussions in the difficulties of routine assessment of the epidemiological situation of fascioliasis because of the lack of veterinarians officially working locally become evident. The recent launching of the new Universidad Pública de El Alto (UPEA) implementing the “Carrera de Medicina Veterinaria y Zootecnia” may help in this endeavour. Unfortunately, different problems are leading to an excessively slow upturn of this new training institution.

Household availabilities have proved their great epidemiological importance in other human hyperendemic areas concerning both liver fluke infection and reinfection of local inhabitants [96,97]. In the Northern Bolivian Altiplano, the inexistence or paucity of systems furnishing for electricity, gas and running treated water at home represent a big problem throughout most of the rural area (Figure 7(Ba)). In the 1990s, home availability of electricity was found only in the population concentrations of El Alto, Viacha, Batallas, Pucarani, Guaqui and Tiwanaku, and smaller villages or communities besides the main roads followed by power lines as those up to Achacachi and Copacabana, or Laja and Tambillo, or down to Patacamaya. Although electricity supply has been pronouncedly improved in recent years, not all dwellings of these localities are provided with this advantage, and several appear uncontrolledly connected. In the deep rural communities, more or less far from the main roads, the lack of electricity in the households is still the rule (Figure 7(Ba)).

Home availability of gas is pronouncedly worse, due to the total lack of gas lines throughout the whole endemic area (Figure 7(Ba)). Therefore, potential control measures by killing metacercariae by heat [13], i.e., cooking food or boiling water, remains restricted to the home use of fire in the kitchen (usually by a peasant stove made with clay) or the use of liquefied gas cans. Dried cattle manure is still used therefore in rural dispersed communities (Figure 8D). The corresponding need of efforts for that purpose, together with given Aymara traditions such as the belief on the inconvenience of boiling natural water, lead to difficulties in the implementation of such in-other-places easy measures.

The biggest problem is, however, the lack of the availability of running treated water inside the households of the rural area (Figure 7(Ba)). Only the larger urban centers have water-treatment plants, and these are limited to chlorination. Nonetheless, although the water may be of bacteriologically acceptable quality when it leaves the plant, there is no assurance that this quality will be maintained until it reaches the consumer, given the shortcomings of the distribution systems [64]. In the rural communities, absence of water availability inside the household and the Aymara traditional belief about the preference for water from a natural source in the field, together with the shared use of the same freshwater collections by humans and animals, suggest a high infection risk through water. All people in the endemic localities recognized having consumed natural water from the field along their life. Although there may be a preference for one type of freshwater collection due to proximity to the household, daily walks to and from the school and along the field transects followed when herding of livestock, underlie the additional use of other types of natural water sources (Figure 7(Bb)). All children (100%) answered similarly in a survey performed from the locality of Cullucachi [82].

Another household deficiency overall the rural zone is the absence of latrine or bathroom inside the dwelling (Figure 7(Bd)). Outside defaecation is commonly practiced (Figure 7(Bc)), mainly in or besides freshwater collections (Figure 8E). Latrines are rarely found in the open field at the service of nearby dispersed household communities (Figure 8F), and several schools have them in their yard. In schools, latrines often lack appropriate management, are not cleaned, and accumulated excreta and dirt are frequently observed, which allows to understand why the children from these schools prefer the open field for defaecation. In a few cases, a latrine is available inside the walled yard of the household (Figure 8I), the resulting man-made excretion canal from the latrine running underneath the external wall and outside connecting with small irrigation-type canals usually presenting lymnaeid populations (Figure 8G,H). Moreover, cesspools and septic tanks are very few throughout the rural endemic area. All in all, this problem is evidently related to the total lack of sewage system in these rural communities.

Defaecation in the open field becomes crucial from the disease transmission and epidemiological points of view, because (i) coprological surveys have demonstrated that Altiplanic inhabitants and mainly children shed liver fluke eggs with their stools in high prevalences and intensities [26,41,42], and (ii) these eggs in human stools are viable [113].

Similar observations about outdoor defaecation were already previously reported from rural localities. A very high proportion of people was highlighted to defaecate in the open field in surveys carried out in communities such as Corapata [81] and Cullucachi [82].

### 3.7. Knowledge of Fasciola and the Disease

Quantifications of the information about the knowledge of the community inhabitants on the liver fluke and the disease it causes, obtained in the ethnographic studies by author observations and personal interviews in the surveys of the localities in which human infection was diagnosed, are shown in Figure 7 and illustrating photographs of crucial aspects are included in Figure 8.

Similarly as for other vector-borne zoonotic parasitic diseases, education of the affected communities is key within a control strategy against fascioliasis [13]. In fascioliasis, however, the many elements of the life cycle of the liver fluke and factors influencing on each stage make it not easy to understand by persons not familiarized with such diseases or with health in general, markedly less by rural communities where individual and family literacy is very low. Relationships of the knowledge, attitudes and traditions of mothers with fascioliasis transmission have already been verified in another Andean human hyperendemic area such as Cajamarca, in Peru [114,115].

In the Northern Bolivian Altiplano, the knowledge of inhabitants about the liver fluke, its snail vector and transmission, the disease it causes in humans and animals, and the infection sources, appears to be very fragmented (Figure 7C). The existence of a vernacular specific name as “talpalako” to refer to the liver fluke in the Aymara language already talks on an old knowledge about the parasite. Indeed, families unavoidably see the parasite adults in the liver of the animals they slaughter when they are infected (Figure 7(Ca)). Hence, the existence of the “talpalako” appears widely recognized by the adult inhabitants in the rural zones and they usually know when it is present in the animals of a given zone or not. Although sometimes the answers telling about all livestock infected pose credibility doubts, the correctness of the answers in places where it is not present could be verified by the absence of lymnaeid-inhabited freshwater collections. The possibility of infection of humans with the same liver fluke which infects the animals, however, does not appear to be so widely assumed (Figure 7(Ca)).

Regarding the transmission, several inhabitants do link the infection of the animals with the freshwater milieu, but knowledge about the involvement of freshwater snails and which kind of snails in the liver fluke transmission is almost inexistent. When performing field in situ training of the women and children in charge for livestock herding (Figure 3F), in the way to allow them to distinguish which freshwater collections are risky for animal and also human infection, by detecting the presence of lymnaeid snail vectors in them, the curiosity faces of the trained Aymaras did not suggest they were correctly understanding that such small, apparently inoffensive snails were at the origin of the disease of their animals and/or children (Figure 7(Cb)).

Whereas the hepatic microhabitat of the fluke is widely known, mainly because they see the parasites in the liver of the animals they slaughter, the involvement of freshwater plants and freshwater from natural collections appears not always correctly assumed by adult Aymaras (Figure 7(Cc)), as in fact this partly contradicts their “Pachamama” century old beliefs. Something similar occurs when relating the symptoms as manifestations of the disease with the presence of the liver fluke in the liver of the ill person. There usually is no conscious relation between the cause and the effects. Symptoms are attributed to punishment by the mother nature according to old beliefs (Figure 7(Cc)).

The paucity of information on disease and disability in the typical highland Aymara rural communities was already early highlighted [71]. The wide reluctance to accept medical assistance has been reported and the reasons for such a response have been analysed [116]. Resorting to herbal remedies according to traditional Aymara medicine also underlie the reluctance in question [117].

Yearly repeated teaching of children at schools, with colour illustrated images in posters, may slowly and gradually change such disease conception. Appropriate efforts to be made by adequately trained local community authorities and also young parents may also help in this endeavour, although very old traditions will evidently make this not an easy and fast task (Figure 7(Cd)). Appropriate message diffusion by radio stations, located in El Alto, in Aymara language, with coverage all over the rural zones up to the Lake Titicaca, and received by very cheap, battery-charged radio devices, may also be used for such purposes. Such kind of radio attempts have already been proposed regarding Altiplanic livestock infection and treatments in the past [81] and has been also suggested for the changing of behaviours related to education and health [118].

Despite the aforementioned confrontation of Aymara beliefs and traditions with modern medical and veterinary care, treatment of livestock is widely practised according to animal owners. On the contrary, measures to avoid animal infection in the field are very rarely observed throughout the rural zones of the endemic area. Similarly occurs with the measures to avoid human infection by taking care of freshwater plant species usually carrying liver fluke metacercariae and drinking or using natural water for food and beverage preparation and washing of fruits, vegetables, roots and tubers and kitchen utensils (Figure 7(Ce)). Difficulties in understanding the relationships between the infection sources and disease consequences, together with Aymara cultural beliefs on the mother nature, no doubt underlie such an apparent nonsense in risk perception.

### 3.8. Behavioural, Traditional, Social and Religious Aspects

**Figure 9:**
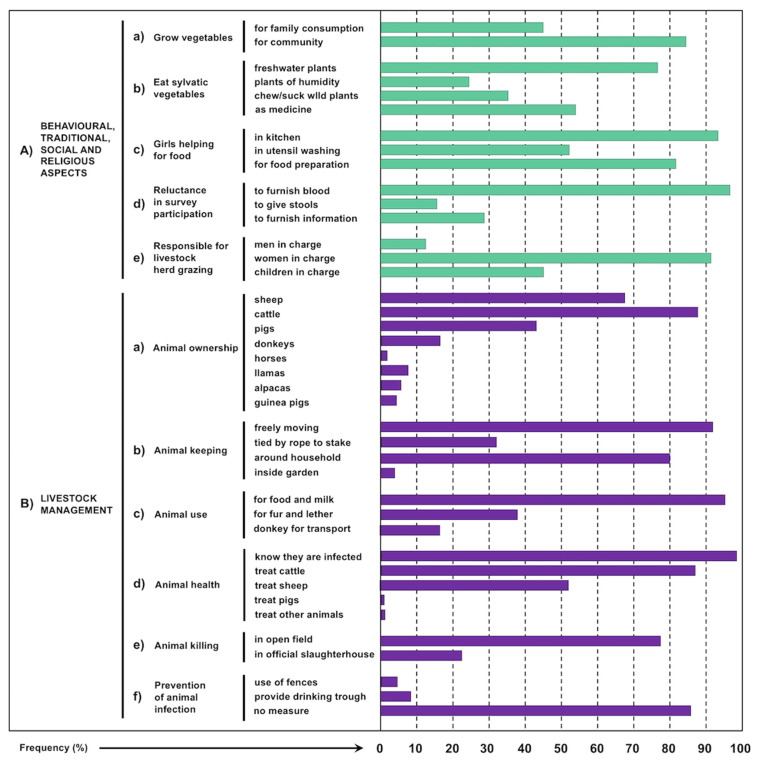
Results (%) of the quantitative analyses of the characteristics of 23 Aymara communities presenting human infection by *Fasciola hepatica*, covering all zones of the human fascioliasis hyperendemic area in the Northern Bolivian Altiplano with transmission foci in each endemic inter-hilly flat corridor or transect, including information obtained in the ethnographic fieldwork, during all seasons and throughout a very long period of 35 years, about aspects linked to fascioliasis transmission and human infection: (**A**) behavioural, traditional, social and religious aspects; (**B**) livestock management.

Other worth mentioning aspects emerged during the field work in the human communities presenting *F. hepatica* infection. Several are related to the crucial bias of the impacts of the disease on age and gender, i.e., higher infection risk for children and women. Quantified results of behavioural, traditional, social and religious aspects are noted in Figure 9A.

The Aymara inhabitants of the Northern Bolivian Altiplano are small-scale farmers and ranchers following a subsistence strategy within a respect to the local agrobiodiversity regarding what is grown and what nature offers. Their diet is composed by the following elements:

- Vegetables they grow in reduced crops (Figure 6H and Figure 8B,C). The very high altitude hostile environment including dry and cold climate play an important limiting factor for agricultural production [119], although elderly Aymara persons still keep valuable knowledge acquired by previous generations about crop strains more resistant to long dry periods [120]. Therefore, crop purpose is mostly to feed families living in communities (Figure 9(Aa)) and whose consumption varies according to the rainy and dry seasons. Seeds and vegetables grown in crops may be stored in a specific room which is strictly in charge of women.
- As complements they use edible tubers and sylvatic plants (with preference for tender leaves) used as condiments and which are consumed fresh and only rarely stocked for short time (Figure 5B–G). Indeed, a wide traditional knowledge on edible sylvatic plants whose leaves, stems and flowers are consumed tender appears in the culture of native ethic groups living in rural communities throughout the Americas since pre-Hispanic times. The additional use of several of these plants as in house medicinal remedies for diseases of the digestive system and liver should be highlighted [121,122]. In the Northern Altiplano, inhabitants are used to consume sylvatic plants, among which the most tender and tasty growing in freshwater and humid environments, whether as food condiments or traditional medicine remedies. The habit to chew and/or suck juicy sylvatic plants, as the stems of “totorillas”, is recognized by many children (Figure 9(Ab)).
- Familiar livestock husbandry allows to obtain fresh meat from the animals they breed and slaughter, above all from cattle, sheep and pigs that are the main three livestock species owned by the families. Donkeys are used for good transport [49]. Llamas and alpacas are very few throughout the endemic area, although several may be seen in the northern part, as in the subcorridor of Peñas and the farms of Belen [50], and meat of llama is sporadically consumed.
- Fish is part of the diet only in the communities and municipalities of zones surrounding the Lake Titicaca (Figure 1 and Figure 2).
- Other agricultural products and industrialized foods are sometimes obtained by exchange in the local markets of the area, exchange activities which appear to gradually increase recently due to the increase in dry periods making family crops difficult, pronounced effort-needing, and less productive [123].

In the life of Aymaras, children are said to daily learn by observing the activities of parents [124]. In this context, girls begin very precociously to learn on food preparation and kitchen activities from the mothers. Aymaras tell about about their daughters being able to know which meal corresponds for every day as early as the age of 7 years (Figure 9(Ac)). This fact may presumably underlie the higher liver fluke infection intensities in girls than in boys found in coprological surveys of schoolchildren [26]. As recently experimentally ascertained, a higher infection risk leads to reinfections which in turn give rise to an increase in egg shedding because of the burden increase caused by the lack of premunition [38]. This also agrees with the highest burdens reported to be more precocious in females than males within a peak in the 7–10 year-old children group, in another human hyperendemic area [96].

During the survey activities performed, a general rejection to blood extraction for the serological diagnosis of fascioliasis was constated almost everywhere in the Northern Altiplano (Figure 9(Ad)). Reference to a fraudulent use of the blood samples to obtain money frequently appears when a serological survey is proposed. In the communities where such rejection was found, a reluctance to treatments and a distrust to all governmental initiatives was sometimes observed in the adult and elderly subjects. In women and girls, this seems to be related to extreme shyness and/or a tendency not to talk about personal or intimate topics. Thus, women and even the girls beyond the age of 10 or 11 may reject the contribution of their own stool samples and furnishing information about their activities at home (Figure 9(Ad)). Such an elusive behaviour has already been reported and deeply analysed regarding health initiatives in general [116].

**Figure 10:**
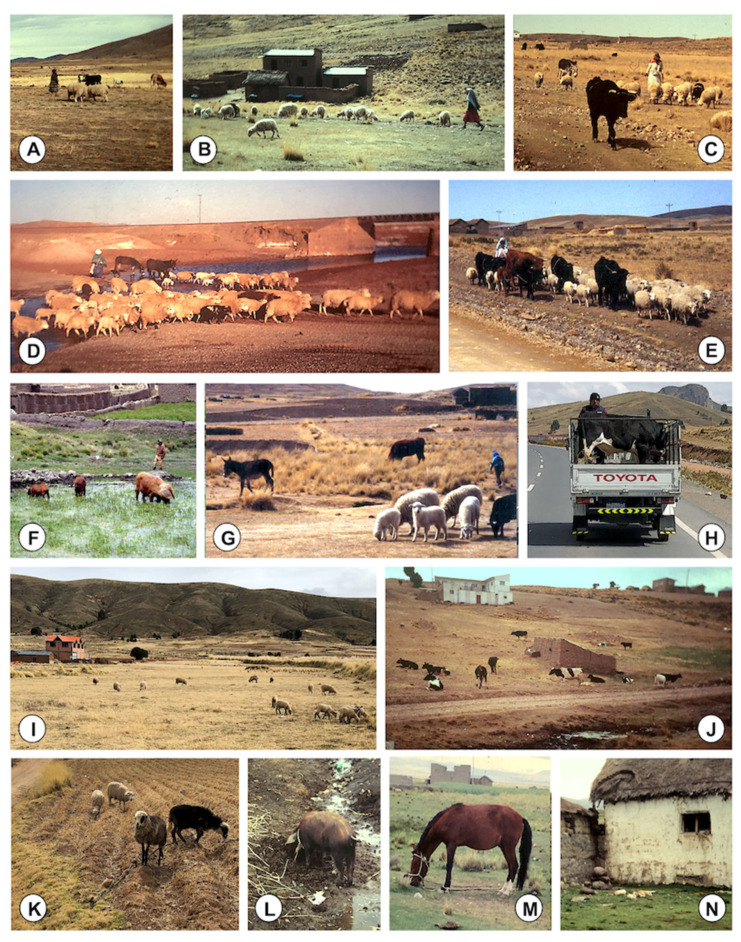
Livestock management: (**A**–**E**) Women are in charge of moving the herds of sheep, cattle, and donkeys, as in Achacachi (**A**), Huarina (**B**,**C**,**E**), and Capiri (**D**); (**F**,**G**) Children undertake responsibilities in livestock management starting at a very early age, as in Pucarani (**F**) and Copancara (**G**); (**H**) Transport of livestock is increasingly carried out with vehicles; (**I**,**J**) Freely running livestock is almost everywhere, as in Lacaya (**I**) and Kajchiri (**J**); (**K**–**M**) Stakes and rope are sometimes used to keep domestic animals in place, as for sheep in Santa Rosa (**K**), pigs in Batallas (**L**), and horses in Viacha (**M**); (**N**) Guinea pigs, locally known as “kuwis” or “cuyes”, are rarely kept by Aymaras in the Northern Altiplano, as in Peñas. Photographs S. Mas-Coma. F modified from Mas-Coma et al., Parasitology, 2018, 145, 1665–1699.

Another behaviour of interest for fascioliasis is the tradition of herding the livestock in the field (Figure 9(Ae)). This activity appears to be usually in charge of the women (Figure 10A–E). Herds include from a relatively few animals up to herds composed by many. Animal species in these herds are mainly sheep, secondarily cattle in fewer number, and sometimes also a very few donkeys. During herding, the activities of women consist of keeping watch on the herd and guide it to pastures and freshwater collections for drinking, a crucial aspect mainly during dry periods. Although other members of the family may also take part in herding, men are seen to be very rarely involved, whereas children do participate more actively even regarding pigs (Figure 10F) and sometimes those in charge are very young children (Figure 10G). Such long daily periods in the field for herding represent a risk because of the opportunities to chew and/or suck juicy sylvatic plants (Figure 5E) or drink water from freshwater collections. Trucks are also progressively used for the transport of animals in the Altiplano in recent times (Figure 10G).

### 3.9. Livestock Management

Quantifications of the data on livestock management of importance regarding fascioliasis transmission and risk of human infection, obtained in the ethnographic studies by author observations and personal interviews in the surveys of the communities in which human infection was diagnosed, are shown in Figure 9B and illustrating photographs of crucial aspects are included in Figure 10.

Domestic animals are the main sources of income for the subsistence of the families in the Northern Bolivian Altiplano, because of the reduced production of crops owing to the extreme environmental conditions limiting plant growth. Thus, the number of animals owned is important in defining the social status of a family in the community (Figure 9(Ba)). Within this subsistence strategy, animals are mainly used for food and milk, secondarily for fur and leather, and the donkey as the key species for good transport (Figure 9(Bc)).

In the endemic area, sheep are the most numerous livestock species, although cattle are more usually seen in field observations (Figure 9(Ba)). The liver fluke prevalences in these two species, together with the viability and infectivity of the *F. hepatica* isolates from Altiplanic sheep and cattle, despite the very high altitude inhospitality of the endemic area, have proved that sheep and cattle should be considered the first and second reservoir species for control priorities [47].

Relatively large numbers of both species are usually seen moving freely throughout the field and besides dwellings (Figure 9(Bb) and Figure 10I,J). The only strategy observed to keep animals controlled in place is by maintaining them tied to a stake with a rope around their neck or head (Figure 9(Bb) and Figure 10K–M). Fences are not used for such a purpose in the Altiplano endemic area.

The pig is the third domestic livestock species in number (Figure 10F,L), although quantitatively at distance from sheep and cattle (Figure 9(Ba)). Experimental studies on the transmission capacity of Altiplanic liver fluke isolates from pigs and field studies on prevalences in the endemic area have shown that the pig is a very efficient reservoir and should be catalogued third for control priorities [48]. This contrasts with past studies referring to the lack of susceptibility, even refractory characteristics, of the pig regarding *F. hepatica* in Europe. Freely moving pigs, including piglets, are usually found in the Altiplano endemic area [48], in several cases controlled in herding activities in the field (Figure 10F), or tied with a rope (Figure 10L), and sporadically also inside walled “patios” or yards surrounding the household (Figure 8I).

The donkey is frequently present and a member of the livestock owned by the rural families (Figure 10C,D,G) throughout the whole of the fascioliasis endemic area of the Northern Altiplano (Figure 9(Ba)). Liver fluke infection of donkeys occurs in the Altiplano, but its transmission capacity has recently demonstrated to be markedly less efficient than in sheep, cattle, and pigs. However, the use of donkeys for good transport represents an evident risk for the spreading of both *F. hepatica* actively with their faeces and *G. truncatula* snails passively attached with mud to their grooves. Consequently, donkeys may play a role in the spread of the disease, from inside one part to another part of the endemic area, as well as to outside of the endemic geographic actual limits [49].

Mules, well known in other very high altitude Andean zones because of their role in the transportation of persons and goods and recently proven to be reservoirs and spreaders of fascioliasis [125], are absent in the Altiplano. Indeed, working mules are useful mainly in difficult rugged mountainous routes and the donkey is more than sufficient for the flatland corridors of the Altiplano. A few horses may sporadically be observed in the Northern Altiplano endemic area (Figure 10M). However, their isolated presence (Figure 9(Ba)) and higher resistance to liver fluke infection when compared with donkeys and mules [125,126,127] indicate that a potential role for the horse as fascioliasis reservoir in the Altiplano should be neglected.

A similar consideration is merited regarding goats. Although goats exist in the Northern Bolivian Altiplano endemic area, they are so few and isolated that their role in the local liver fluke transmission should also be neglected, despite the well known reservoir capacity of this livestock species.

Llamas and alpacas are present in the Northern Altiplano (Figure 9(Ba)) and are known definitive hosts for *F. hepatica*, but a potential role as reservoirs has been ruled out, as recently proved by experimental and field studies [50]: (i) the liver fluke transmission capacity of llama isolates is not sufficiently efficient; (ii) they do not show liver fluke infection in the Altiplano because they are present in zones where dryness, absence of freshwater collections, and higher altitudes do not allow lymnaeids to be present; (iii) the dung-pile defaecation behaviour of these camelids, always far from freshwater collection, does not allow liver fluke eggs shed by these animals to reach water and infect lymnaeids [50]. Infection of alpacas has been reported from the Northern Bolivian Altiplano, but this only concerned the farm of Belen [76], which is outside the human endemic area. Field studies inside the human endemic area were never able to find *F. hepatica* infection in these camelids. Therefore, control measures for South American camelids are not needed in the Northern Altiplano [50].

Guinea pigs are domestic rodents traditionally owned by Aymaras and also Quechuas inhabiting the high Andean altitude zones of South American countries. Although not frequent (Figure 9(Ba)), they can sometimes be seen besides the Aymara households in the Northern Bolivian Altiplano (Figure 10N). Studies on this domestic rodent showed that they may be neglected regarding a potential role as reservoirs of the liver fluke in this endemic area [128]. A similar consideration might be mentioned concerning the domestic rabbit, very rare in the Altiplano endemic area [128].

Appropriate field surveys already proved that neither sylvatic lagomorphs, including rabbits and hares, nor wild herbivorous rodents, including several species of Cricetidae but also Caviidae and Muridae, are infected by the liver fluke in the Northern Bolivian Altiplano hyperendemic area [128].

Adult subjects inhabiting the rural zones of the Altiplano which include transmission foci usually answer positively to the question about liver fluke infection of the animals of their local zone (Figure 9(Bd)). In these cases, they also speak about the yearly treating of their animals, referring mainly to cattle and secondarily sheep, although of course this may depend on the availability of the needed funds by the family owner. In no case did they refer to treating the pigs, nor other animal species (Figure 9(Bd)). Interestingly, the absence of transmission foci could be verified in zones where the local inhabitants answered that their animals were not infected by the “talpalako”.

In treatments, Altiplanic Aymaras give priority to cattle, which is related to the seasonal very pronounced decrease in milk production when the liver fluke infection rates in cattle are higher, e.g., in August [59]. The economic impact of the liver condemnation in cattle infected by *F. hepatica* has recently also been ascertained in altitude areas of the neighbouring Peru [129].

**Figure 11:**
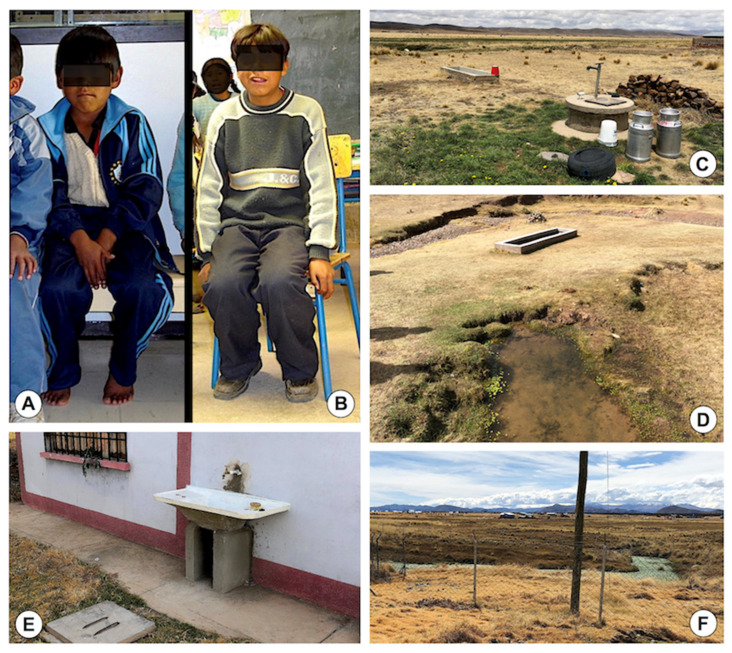
Control measures: (**A**,**B**) A 13-year old boy weighing only 26 kg and shedding 384 *F. hepatica* eggs per gram of faeces on 23 June 2008 in Hospital del Niño of La Paz (**A**) pronouncedly improved in development to 41 kg in 12 November 2008 in Huacullani when coprologically negative after treatments in the yearly preventive chemotherapy programme (**B**); (**C**) Well and recently constructed drinking-trough for livestock, in Challapata; (**D**) Artificial drinking-trough for livestock constructed besides freshwater collection inhabited by lymnaeid vectors and surrounded by animal stools, in Santa Rosa; (**E**) External faucet and basin in front of a health centre, in Huacullani; (**F**) Fences installed around transmission focus inhabited by lymnaeid vectors besides hyperendemic village, in Huacullani. (**A**,**B**) Photographs R. Angles; (**C**–**F**) Photographs S. Mas-Coma; E and F modified from Bargues et al., Parasit. Vectors 2020, 13, 171.

The past high impact and mortality rates caused by *F. hepatica* in the 1970s, estimated to have been no less than 15–25% annually [76], also include treatments for sheep. Indeed, rural families could have up to 200 and 250 sheep [81], whereas nowadays such numerous sheep herds are rarely seen (Figure 10D). Fascioliasis prevalence in sheep did not show big differences depending on the number of sheep owned (small owners: 1–20 sheep; medium: 21–50; big: >50) [130]. The importance of treatments in Northern Altiplano is crucial, because there is absolutely no tradition to use other animal infection prevention measures as fences or drinking throughs (Figure 9(Bf)), although a few given communities have very recently begun to realize the appropriateness of such initiatives (Figure 11C–F).

In the Northern Altiplano, local traditions, reluctance to pay, and fear of potential inspections lead to the uncontrolled slaughtering of the animals by the owners (Figure 9(Be)). Numerous families reunite one day of the week to simultaneously kill their pigs at the river besides the locality of Batallas [48]. The absence of official slaughterhouses throughout the rural area and the long distance to the official slaughterhouse of El Alto underlie this problem due to the lack of the adequate veterinary inspections of slaughtered animals.

## 4. Conclusions

The health improvements obtained by preventive chemotherapy by means of the yearly massive treatments with a triclabendazole mono-dose indicate the appropriateness of this action (Figure 11A,B). The present multidisciplinary One Health initiative highlights the different aspects needing interventions to decrease infection and re-infection risks in between the yearly treatments. Additionally, the results furnish the baseline for the establishment of priorities, which may help authorities and health-responsible actors to decide how and where is better to apply the funds available to obtain faster impact on the disease.

The present multidisciplinary analysis of all the characteristics of the lifestyle of the inhabitants of the Northern Bolivian Altiplano human hyperendemic area, related to human infection or influence on the transmission and epidemiology of fascioliasis, is the widest and deepest study of this kind ever performed. This has been feasible thanks to around 35 years of research efforts which have allowed us to obtain unique and extensive knowledge on all aspects of fascioliasis in this area. In no other areas of human fascioliasis is such a multidisciplinary knowledge available at present. This has allowed us to carry out the analysis from both directions, i.e., for the appropriate interpretation of the epidemiological importance of each of the social aspects, behaviour, and traditions in the human communities affected, but also to look for which of these could explain the given fascioliasis peculiarities in this area.

The results obtained highlight the difficulties for fascioliasis control and preventive measures in areas where the inhabitants follow century-old behaviours, traditions, and beliefs. It may take generations until the needed aspects are able to change. However, modifying the ethnic identity of the Aymaras is not the way. Our experience suggests that the best way is trying for a complementarity between the “Pachamama” beliefs and modern medical parasitology inputs. Reaching this will require long efforts of education, in which active participation of the leaders of the communities should be looked for. Experiences along this 35-year initiative have already proved that this is feasible, mainly by a first implementation in a selected community, and subsequently convince other communities to follow suit based on the success obtained in the first community as example.

Infrastructure improvements should be undertaken by the national government, La Paz department authorities, and the local leaders. Sufficient networks to provide potable running water and electricity to communities throughout the rural endemic area are key. In such endeavours, the recently improved paved inter-provincial roads up to Guaqui in the third corridor and similarly ongoing with another up to Huacullani in the second corridor are very welcome, although neighbourhood connections still rely on dirt roads. The paved roads will allow for an easier and faster exchange with the cities of El Alto and La Paz, and also facilitate later electrical wiring and water canalizations. The affordability of television at home would undoubtedly accelerate the process.

This study is expected to be useful for human endemic areas in other countries as a model to follow for the control of fascioliasis. It should be considered, however, that human fascioliasis is highly heterogeneous in transmission and epidemiology [13], and that, consequently, the very high altitude features of the endemic area and the Aymara ethnic peculiarities should be taken into account when extrapolating to other endemic areas of completely different characteristics to avoid misinterpretations.

## Figures and Tables

**Table 1:** Edible aquatic and semi-aquatic plants most frequently present in fascioliasis freshwater foci in the Northern Bolivian Altiplano and consumed by Aymara inhabitants.

Family	Species	Aymara Name *	Local Spanish Name *	Presence in 30 Freshwater Collections Inhabited by lymnaeids	
				No.	Percentage
**Cyanophyta**					
Nostocales	*Nostoc sphaericum*	Llaytha	Cochayuyo Cushuro Alga parda	3	10%
**Monocotyledonea**					
Cyperaceae	*Schoenoplectus californicus* spp. *tatora*	Chullu Matara	Tallo de totora	3	10%
		Sakha Matara	Bulbo-raiz de totora	3	10%
	*Eleocharis* spp.	Joskosko	Totorilla	20	66.7%
	*Eleocharis albabracteata*	Joskosko	Totorilla	1	3.3%
Juncaceae	*Juncus* spp.	Joskosko	Totorilla	19	63.3%
	*Juncus andicola*	Joskosko	Totorilla	1	3.3%
	*Juncus bufonius*	Joskosko	Totorilla	2	6.7%
	*Juncus ebracteatus*	Joskosko	Totorilla	11	36.7%
	*Juncus* cf. *microcephala*	Joskosko	Totorilla	1	3.3%
**Dicotyedonea**					
Juncaginaceae	*Lilaea* spp.	Joskosko	Totorilla	14	46.7%
Cruciferae	*Rorippa* (= *Nasturtium*) *nana*	Okororo	Berro	1	3.3%
	*Rorippa* (= *Nasturtium*) *nasturtium-aquaticum*	Okororo	Berro	1	3.3%
Umbelliferae	*Hydrocotyle ranunculoides*	Okororo	Berro	21	70.0%
Scrophulariaceae	*Mimulus glabratus*	Okororo	Berro Flor de mono	9	30.0%
Compositae	*Cotula mexicana*	Okororo ^	Berro ^ Botones de latón mexicano	18	60.0%
	*Taraxacum* spp.	Qhanapaku	Diente de león	6	20.0%
	*Taraxacum officinale*	Qhanapaku	Diente de león	2	6.7%

* Aymara names and local Spanish names do not distinguish at species level. ^ *C. mexicana* may also be confused within the wide term of okororo or berro by children.

**Table 2:** Provinces and municipalities affected by fascioliasis in the Northern Bolivian Altiplano, showing the number of transmission foci studied and of human localities with infected subjects ethnographically analysed and respective prevalence reported.

Province	Municipality	Human Population * Proyections for Years		No. Transmission Foci Studied **	Human Localities with Infected Subjects		Local Human Prevalences ^$^	
		2012	2022		Ethnographically Analysed **	Other Localities Reported ^	E.V.	Mean
**URBAN ZONE**								
MURILLO	La Paz	845,719	956,732	0	0	0	(0) ^&^	0
	El Alto	916,434	1,109,048	0	0	1	0.2	0.2
	**TOTAL URBAN**	1,762,153	2,065,780	0	0	1	0.0–0.2	0.1
**RURAL ZONE**								
MURILLO	Achocalla	17,869	19,995	4	2	0	5.3–9.5	7.4
	TOTAL MURILLO	1,780,022	2,085,775	4	2	0	0.2–9.5	5.0
OMASUYOS	Achacachi	39,763	35,964	2	1	0	1.1–6.5	3.8
	Santiago de Huata	8171	7921	0	0	0	0	0
	Chua Cocani	4965	5503	2	1	0	1.1–3.6	2.35
	Huatajata	4001	4579	0	0	0	0	0
	Huarina	7013	7532	3	1	0	6.3	6.3
	TOTAL OMASUYOS	63,913	61,499	7	3	0	1.1–6.5	3.72
LOS ANDES	Batallas	15,952	14,892	15	2	4	12.6–72.0	48.78
	Puerto Perez	7509	7670	7	4	0	7.0–30.3	15.55
	Pucarani	24,679	24,795	11	7	2	2.7–28.2	15.82
	Laja	19,343	17,670	6	1	1	5.9–47.7	26.8
	TOTAL LOS ANDES	67,483	65,027	39	14	7	2.7–72.0	26.28
INGAVI	Taraco	6336	6491	4	0	0	0	0
	Tiwanaku	10,728	9908	6	3	0	7.3–38.2	23.65
	Guaqui	7113	7672	0	0	0	0	0
	Desaguadero	8768	7394	0	0	0	--	--
	Jesus de Machaca	13,270	12,067	0	0	0	--	--
	Viacha	89,772	85,703	4	1	1	1.3	1.3
	TOTAL INGAVI	135,987	129,235	14	4	1	1.3–38.2	20.46
AROMA	Collana	4373	3900	0	0	0	--	--
	Colquencha	8999	8157	0	0	0	--	--
	Calamarca	11,599	10,836	0	0	0	--	--
	Ayo Ayo	7914	8359	2	0	0	--	--
	Patacamaya	21,566	22,303	1	0	0	--	--
	TOTAL AROMA	54,451	53,555	3	0	0	--	--
	**TOTAL RURAL**	339,703	329,311	67	23	8	1.1–72.0	14.46

* = according to INE Bolivia [57]; ** = present study; ^ = reviewed in Mas-Coma et al. [27]; ^&^ = isolated cases diagnosed in hospitals; ^$^ = coprologically diagnosed (i.e., subjects shedding fasciolid eggs); -- = no surveys performed.

**Table 3:** Gender comparison in the analysis (Fisher’s exact test) of positive answers on vegetable food habits by inhabitants of the localities of Cutusuma and Tauca in the Northern Bolivian Altiplano human fascioliasis hyperendemic area, potentially related to fascioliasis transmission and human fascioliasis. Significant differences highlighted in bold.

Vegetable Consumption		Positive Answers in Total Participant Subjects No./%			Positive Answers in Infected Subjects No./%		
Aymara name	Spanish translation *	Males	Females	P	Males N = 20	Females N = 22	P
**CUTUSUMA** 194 participants: 100 males and 94 females							
Chullu	tallo de totora	**81/81.00%**	**88/93.61%**	**0.0100**	17/85.00%	20/90.91%	ns
Joskosko	totorillas	**51/51.00%**	**63/67.02%**	**0.0287**	13/65.00%	14/63.63%	ns
Okororo	berros	37/37.00%	33/35.11%	ns	**9/45.00%**	**3/13.64%**	**0.0400**
Sakha	bulbo de totora	64/64.00%	72/76.70%	ns	13/65.00%	14/63.63%	ns
Llaytha	alga parda	63/64.00%	63/67.02%	ns	13/65.00%	15/68.18%	ns
**TAUCA** 100 participants: 52 males and 48 females							
Chullu	tallo de totora	45/86.54%	43/89.58%	ns	-	-	-
Joskosko	totorillas	16/30.77%	21/43.75%	ns	-	-	-
Okororo	berros	**22/42.31%**	**30/62.50%**	**0.0483**	-	-	-
Sakha	bulbo de totora	28/53.85%	23/47.92%	ns	-	-	-
Llaytha	alga parda	16/30.77%	10/20.83%	ns	-	-	-

* = for corresponding species names see Table 1. ns = not significant.

**Table 4:** Age group comparison (2–15-year-old children vs. 25–76-year-old adult subjects) in the analysis (Fisher’s exact test) of positive answers on vegetable food habits by inhabitants of the localities of Cutusuma and Tauca in the Northern Bolivian Altiplano human fascioliasis hyperendemic area, potentially related to fascioliasis transmission and human fascioliasis. Significant differences highlighted in bold.

Vegetable Consumption		Positive Answers in Total Participant Subjects No./%			Positive Answers in Infected Subjects No./%		
Aymara name	Spanish translation *	Children	Adults	P	Children N = 39	Adults N = 3	P
**CUTUSUMA** 194 participants: 135 children and 59 adults							
Chullu	tallo de totora	115/85.19%	54/91.52%	ns	34/87.18%	3/100%	ns
Joskosko	totorillas	78/57.78%	36/61.02%	ns	24/61.54%	3/100%	ns
Okororo	berros	**42/31.11%**	**28/47.46%**	**0.0350**	10/25.64%	2/66.66%	ns
Sakha	bulbo de totora	**88/65.19%**	**48/81.36%**	**0.0268**	24/61.54%	3/100%	ns
Llaytha	alga parda	**81/60.00%**	**45/76.27%**	**0.0337**	25/64.10%	3/100%	ns
**TAUCA** 100 participants: 72 children and 28 adults							
Chullu	tallo de totora	63/86.54%	25/89.28%	ns	-	-	-
Joskosko	totorillas	23/30.77%	14/50.00%	ns	-	-	-
Okororo	berros	**32/42.31%**	**20/71.43%**	**0.0249**	-	-	-
Sakha	bulbo de totora	35/53.85%	16/57.14%	ns	-	-	-
Llaytha	alga parda	18/30.77%	8/28.57%	ns	-	-	-

* = for corresponding species names see Table 1. ns = not significant.

**Table 5:** Multivariate logistic regression analysis of different data obtained from children and their answers on vegetable consumption in the locality of Cutusuma in the Northern Bolivian Altiplano human fascioliasis hyperendemic area, and regression coefficients [Exp(B) = RR = relative risk of fascioliasis determined by coprodiagnosis] with significance in two models including presence/absence of liver fluke infection as dependent variable: **Model 1**, including gender, age, and weight of children as independent variables; **Model 2**, including gender, age, and weight of children, plus positive answers on consumption of the five listed vegetables as independent variables.

		Model 1		Model 2	
		Coefficient	Significance	Coefficient	Significance
**Data from 99 Children**					
Gender (females) N/%	49/49.5%	1.826	ns	1.939	ns
Age (years old) Average ± SD (min-max)	8.6 ± 2.9 (2–15)	1.455	0.047	1.527	0.046
Weight (kg) Average ± SD (min-max)	24.49 ± 7.5 (8–53)	0.856	0.036	0.853	0.049
Fascioliasis infection N/%	39/39.4%				
Vegetable Consumption *					
Chullu/tallo de totora N (%)	86/86.9%			0.749	ns
Joskosko/totorillas N (%)	55/55.6%			1.344	ns
Okororo/berros N (%)	30/30.3%			0.596	ns
Sakha/bulbo de totora N (%)	63/63.6%			0.601	ns
Llaytha/alga parda N (%)	55/55.6%			1.641	ns

* = for corresponding species names see Table 1. ns = not significant.

## Data Availability

The datasets generated for this study are available on request to the corresponding author.
